# Possible involvement of zinc transporter ZIP13 in myogenic differentiation

**DOI:** 10.1038/s41598-024-56912-7

**Published:** 2024-04-12

**Authors:** Masaki Shoji, Takuto Ohashi, Saki Nagase, Haato Yuri, Kenta Ichihashi, Teruhisa Takagishi, Yuji Nagata, Yuki Nomura, Ayako Fukunaka, Sae Kenjou, Hatsuna Miyake, Takafumi Hara, Emi Yoshigai, Yoshio Fujitani, Hidetoshi Sakurai, Heloísa G. dos Santos, Toshiyuki Fukada, Takashi Kuzuhara

**Affiliations:** 1 Laboratory of Biochemistry, Faculty of Pharmaceutical Sciences, Tokushima Bunri University, 180 Nishihamahouji, Yamashirocho, Tokushima-City, Tokushima 770-8514 Japan; 2 Laboratory of Molecular and Cellular Physiology, Faculty of Pharmaceutical Sciences, Tokushima Bunri University, 180 Nishihamahouji, Yamashirocho, Tokushima-City, Tokushima 770-8514 Japan; 3 Laboratory of Developmental Biology and Metabolism, Institute for Molecular and Cellular Regulation, Gunma University, Maebashi-City, Gunma Japan; 4 Center for iPS Cell Research and Application (CiRA), Kyoto University, Kyoto-City, Kyoto Japan; 5 Serviço Genética Médica, Hospital S. Maria, Lisboa, Portugal

**Keywords:** Biochemistry, Cell biology, Developmental biology, Stem cells, Diseases, Molecular medicine, Pathogenesis

## Abstract

Ehlers–Danlos syndrome spondylodysplastic type 3 (EDSSPD3, OMIM 612350) is an inherited recessive connective tissue disorder that is caused by loss of function of SLC39A13/ZIP13, a zinc transporter belonging to the Slc39a/ZIP family. We previously reported that patients with EDSSPD3 harboring a homozygous loss of function mutation (c.221G > A, p.G64D) in *ZIP13* exon 2 (*ZIP13*^*G64D*^) suffer from impaired development of bone and connective tissues, and muscular hypotonia. However, whether ZIP13 participates in the early differentiation of these cell types remains unclear. In the present study, we investigated the role of ZIP13 in myogenic differentiation using a murine myoblast cell line (C2C12) as well as patient-derived induced pluripotent stem cells (iPSCs). We found that *ZIP13* gene expression was upregulated by myogenic stimulation in C2C12 cells, and its knockdown disrupted myotubular differentiation. Myocytes differentiated from iPSCs derived from patients with EDSSPD3 (EDSSPD3-iPSCs) also exhibited incomplete myogenic differentiation. Such phenotypic abnormalities of EDSSPD3-iPSC-derived myocytes were corrected by genomic editing of the pathogenic *ZIP13*^*G64D*^ mutation. Collectively, our findings suggest the possible involvement of ZIP13 in myogenic differentiation, and that EDSSPD3-iPSCs established herein may be a promising tool to study the molecular basis underlying the clinical features caused by loss of ZIP13 function.

## Introduction

Zinc is an essential trace element and its homeostasis is tightly regulated mainly by two types of zinc transporters: Solute carrier 39A (SLC39A)/zrt and irt-like proteins (ZIPs) and solute carrier 30A (SLC30A)/zinc transporters (ZnTs)^1^. SLC39As/ZIPs transport zinc into the cytosol from the extracellular environment or intracellular organelles, while SLC30As/ZnTs transport zinc in the opposite direction^1^. Recent studies have uncovered that these transporters are involved in many cellular and signaling events, such as growth factor-, cytokine-, and transcription factor-mediated signaling^2^ and their dysfunction has been noted in several human diseases^2^, indicating that zinc transporters could be considered potential therapeutic targets^3^.

SLC39A13/ZIP13 is a representative ZIP transporter that is associated with human diseases. Namely, Ehlers–Danlos syndrome spondylodysplastic type 3 (EDSSPD3, OMIM 612350)^4–7^ is caused by homozygous mutations in *SLC39A13*/*ZIP13* gene and is characterized by severe connective tissue impairments, such as short stature, skeletal dysplasia, spine deformity, fragile skin, and hypodontia. This is a relatively rare disease and approximately ten individual patients have been reported so far^6,7^. We previously reported two siblings with EDSSPD3 harboring a homozygous point mutation (c.221G > A, p.G64D) in exon 2 of *ZIP13* (*ZIP13*^*G64D*^)^4^, whose clinical features highly correlated with the phenotype of *Zip13*-deficient (KO) mice^4^. *ZIP13*^*G64D*^ protein is readily degraded via the valosin-containing protein-linked ubiquitin–proteasome pathway, which is attributed as the main cause of EDSSPD3^8^; i.e., loss of functional ZIP13 protein is the molecular mechanism underlying the pathogenesis of EDSSPD3. Patients with EDSSPD3 suffer from muscular symptoms including muscular hypotonia, reduction of muscular strength^4^ and myopathy^9^, a wide spectrum of muscular diseases comprising muscle weakness, muscle hypotonia, atrophies and/or myalgias, muscle stiffness, and cramps^10^. Myopathies are thought to be caused by perturbations in myogenesis as a result of functional abrogation of myogenic regulatory factors (MRFs) such as myogenic differentiation 1 (MyoD)^11^, myogenin^12^, and myogenic factor 5 (Myf5)^13^. Although the molecular mechanism through which ZIP13 regulates myogenesis remains to be elucidated, it can be hypothesized that ZIP13 regulates the expression and/or functions of MRFs during myogenic differentiation.

Human induced pluripotent stem cells (hiPSCs) are well-recognized as beneficial tools that present unique research opportunities not only in regenerative medicine but also in drug discovery as an approach to treat intractable and rare diseases^14^. Consistently, hiPSCs have been used for treatment of Duchenne muscular dystrophy (DMD)^15–17^, Miyoshi myopathy (MM)^18,19^, and Pompe disease^20^. In vitro disease models of skeletal muscles using iPSCs have been established by ectopic introduction of MyoD^21^, and these patient-derived iPSCs can be further differentiated into cells that exhibit clinical features of diseases, such as impairment of dystrophin expression, Ca^2+^ influx, and contractile performance^15,17,19^; thus, patient-derived iPSCs can be used to establish in vitro disease models. However, to the best of our knowledge, there are no reports on the generation and application of patient-derived iPSCs for EDSSPD3. Therefore, establishment of iPSCs derived from patients with EDSSPD3 (EDSSPD3-iPSCs) will provide opportunities to elucidate the pathogenic mechanisms underlying the loss of ZIP13 functions, and can potentially have clinical and pharmaceutical applications.

Herein, we report that ZIP13 is involved in myogenic differentiation. We found that *ZIP13* gene expression was upregulated during myogenic differentiation and its knockdown resulted in failed myogenic differentiation in C2C12 mouse myoblast cells. Additionally, we established EDSSPD3-iPSCs that successfully differentiated into myoblasts, and further demonstrated that the EDSSPD3-iPSC-derived myocytes exhibited attenuated expression of myogenic differentiation markers, which was restored by genetic editing to correct the pathogenic mutation, indicating the possible involvement of ZIP13 in myogenic differentiation. Therefore, our findings suggest that ZIP13 may be a potential novel MRF that regulates normal myogenesis. Further, EDSSPD3-iPSCs have applications not only in the elucidation of mechanisms underlying ZIP13-mediated biological events, but also in regenerative studies and pharmaceutical applications as a treatment approach for EDSSPD3.

## Methods

### Culture and myogenic differentiation of C2C12 cells

**Figure 1:**
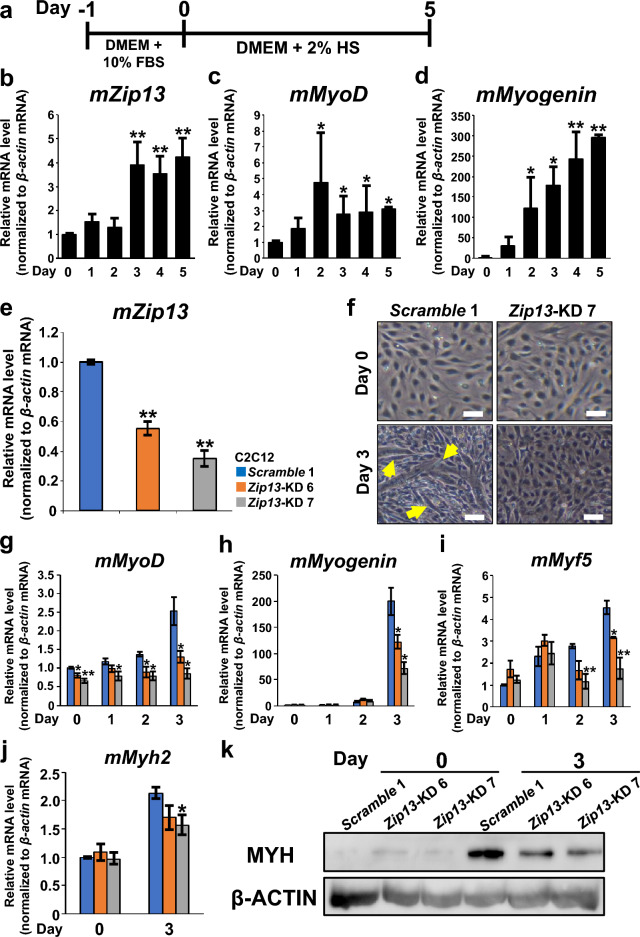
ZIP13 is required for myogenic differentiation of C2C12 cells. (**a**) Schematic of the protocol for horse serum (HS)-induced myogenic differentiation of C2C12 cells. C2C12 cells were cultured for 24 h in Dulbecco's modified eagle medium (DMEM) containing 10% fetal bovine serum (FBS); thereafter, the medium was changed to DMEM containing 2% HS and cultured for 5 days. The cells were harvested at the indicated time points for the indicated analyses. (**b**–**d**) Gene expression profiles of HS-induced *mZip13* (**b**) and myogenic differentiation markers, *mMyoD* (**c**) and *mMyogenin* (**d**), (n = 3 each) analyzed by qPCR. C2C12 cells were harvested at the indicated time points. Data are presented as the mean ± standard error of mean (SEM) of three independent experiments. *P < 0.05 and **P < 0.01 relative to the data on Day 0. (**e**) The expression of *Zip13* mRNA in *Scramble* 1 and two clones of *Zip13*-KD (clone 6 and 7) of C2C12 cells (n = 4 each), developed using the *Zip13* shRNA plasmid was examined by qPCR. Data are presented as the mean ± SEM of three independent experiments. **P < 0.01 relative to the values of *Scramble* C2C12 cells. (**f**–**k**) *Scramble* and two clones of *Zip13*-KD C2C12 cells (6 and 7) were differentiated into skeletal muscle cells by HS treatment as described in Fig. 1a. (**f**) Microscopic evaluation of the morphological changes in *Scramble* and *Zip13*-KD C2C12 cells (clone 7) on Days 0 and 3 of myogenic differentiation. The yellow arrows indicate myotube formation. Scale bar, 50 μm. (**g**–**i**) Gene expression profiles of *mMyoD* (**g**), *mMyogenin* (**h**), *mMyf5* (**i**), and *mMyh2* (**j**) (n = 4 each) in *Zip13*-KD C2C12 cells during myogenic differentiation was analyzed by qPCR at the indicated time points (Days 0–3). Data are presented as the mean ± SEM of three independent experiments. *P < 0.05 and **P < 0.01 versus *Scramble* C2C12 cells. (**k**) MYH protein expression in *Scramble* and two clones of *Zip13*-KD C2C12 cells on Days 0 and 3 of myogenic differentiation were analyzed by western blotting. All data were collected from 3–4 independent experiments.

C2C12 cells were cultured and maintained in Dulbecco’s modified Eagle complete medium (DMEM; WAKO, Osaka, Japan) supplemented with 10% fetal bovine serum (FBS; Thermo Fisher Scientific, Waltham, MA, USA) and antibiotic–antimycotic mixture containing 100 units/mL penicillin, 100 µg/mL streptomycin, and 0.25 µg/mL amphotericin B (Nacalai Tesque, Kyoto, Japan) at 37 °C in the presence of 5% CO_2_. Myogenic differentiation of C2C12 cells was performed as described previously^22^. A schematic depiction of the myogenic differentiation protocol of C2C12 cells is shown in Fig. 1a. Briefly, C2C12 cells were seeded at a density of 1 × 10^5^ cells/dish in 6 cm-dish with 4 mL of DMEM (WAKO) supplemented with 10% FBS (Thermo Fisher Scientific) and the antibiotic–antimycotic mixture at 37 °C in the presence of 5% CO_2_. The medium was replaced the next day with DMEM containing 2% horse serum (Sigma-Aldrich, St. Louis, MO, USA) and the antibiotic–antimycotic mixture. After 5 days of incubation, myogenic differentiation was evaluated by microscopic observation based on the formation of myotubes.

### Establishment of *Zip13*-knockdown (KD) C2C12 cells

Either mouse *Zip13*-shRNA or non-specific (*Scramble*) DNA (Supplementary Table S1) were inserted into the pSUPER.retro.puro vector plasmid (OligoEngine, Seattle, WA, USA) according to the manufacturer’s instructions to generate *Zip13*-shRNA or *Scramble* plasmids. C2C12 cells were transfected with either *Zip13*-shRNA or *Scramble* plasmids using Lipofectamine LTX (Thermo Fisher Scientific) according to the manufacturer’s instructions and cultured in a medium containing 5 µg/mL puromycin for 48 h. Cells were then collected, and the KD efficiency of *Zip13* mRNA was examined by reverse transcription quantitative real-time polymerase chain reaction (RT-qPCR) analysis. The remaining cells were seeded by limiting dilution to generate a monoclonal cell line for both *Zip13*-KD and *Scramble* C2C12 cells. We examined *Zip13* mRNA expression in the twelve monoclonal cell lines and selected two clones (clone 6 and 7) based on the KD efficiency of *Zip13*, and four clones of *Scramble* C2C12 cells (clone number: 1, 5, 9, and 13) were used as the experimental control.

### Ethical approval

All experimental procedures used in the study were approved by the Ethics Committee of Tokushima Bunri University (approval number R2-19 and R3-16) and performed in accordance with the relevant guidelines and regulations. Written informed consent was obtained from the two patients with EDSSPD3 (one female and one male: siblings), harboring a homozygous point mutation *ZIP13*^*G64D*^,^4^ who were included in the present study, for the use of their human dermal fibroblasts (HDFs).

### Culture of HDFs

Healthy 1 (H1, female, Caucasian, 36 years old) or healthy 2 (H2, male, Caucasian, 49 years old) HDFs were purchased from Cell Applications, Inc. (San Diego, CA, USA). HDFs from patient 1 (EDSSPD3-P1, female) and patient 2 (EDSSPD3-P2, male) with EDSSPD3^4,8^ were isolated. HDFs were cultured and maintained in DMEM (WAKO) supplemented with 10% FBS (Hyclone Laboratories, South Logan, UT, USA), 1 × GlutaMAX (Thermo Fisher Scientific), and 100 units/mL penicillin and 100 µg/mL streptomycin (P/S; Thermo Fisher Scientific) at 37 °C in the presence of 5% CO_2_.

### Generation of hiPSCs using an episomal vector

hiPSCs were generated in an integration-free manner with episomal plasmid vectors (http://www.cira.kyoto-u.ac.jp/e/research/protocol.html)^23^. Briefly, H1-, H2-, EDSSPD3-P1-, and EDSSPD3-P2-DFs were cultured in DMEM supplemented with 10% FBS (Hyclone Laboratories), 1 × GlutaMAX (Thermo Fisher Scientific), and P/S (Thermo Fisher Scientific). Episomal plasmid vectors pCXLE-hOCT3/4-shp53-F, pCXLE-hUL, and pCXLE-hSK, which express human OCT3/4 and shRNA against p53, human L-MYC and LIN28, and human SOX2 and KLF4, respectively, were purchased from Addgene Inc. (Watertown, MA, USA) and used for generation of integration-free hiPSCs. Three micrograms of the episomal plasmid mixture (1 µg of each plasmid) were electroporated into 3 × 10^5^ HDFs using the Neon transfection system (MPK5000; Thermo Fisher Scientific). The electroporation conditions were 1650 V, 10 ms, and 3 time pulses. The cells were cultured for 7 days after transduction at 37 °C in the presence of 5% CO_2_, and 5 × 10^4^ cells were replated onto mitomycin-C (Kyowa Hakko Kirin, Tokyo, Japan)-treated SNL76/7 (SNL) feeder cell (DS pharma Biomedical, Osaka, Japan) layer in 60-mm dishes. The next day, the culture medium was replaced with primate embryonic stem cell (ESC) medium (ReproCELL, Kanagawa, Japan) supplemented with 4 ng/mL recombinant human basic fibroblast growth factor (WAKO) and P/S (Thermo Fisher Scientific). Colonies were generated 20–30 days after plating, and colonies with morphology similar to hESCs were selected and grown on mitomycin-C-treated SNL feeder cell layer.

### Feeder-free culture of hiPSCs

The selected iPSCs were maintained under feeder-free condition in an uncoated manner using laminin fragments as described previously^24^. Briefly, iPSC colonies derived from HDFs of healthy individuals (H1 and H2) or patients with EDSSPD3 (P1 and P2) were dissociated with a detachment solution comprising 0.5 × TrypLE select (Thermo Fisher Scientific) and 0.75 mM EDTA in Dulbecco's phosphate-buffered saline (DPBS) (−) (Nacalai Tesque) for 10 min at 25 °C after the removal of SNL feeder cells using CTK solution (2.5% trypsin [Thermo Fisher Scientific], 1 mg/mL collagenase IV [Thermo Fisher Scientific], 0.1 M CaCl_2_, and 20% knockout serum replacement [KSR; Thermo Fisher Scientific] in H_2_O). Colonies consisting of single cells were suspended in StemFit medium (AK02N; Ajinomoto, Tokyo, Japan) supplemented with 10 µM Y27632 (WAKO), Rho-associated, coiled-coil containing protein kinase inhibitor, and P/S (Thermo Fisher Scientific). The cells were then seeded at a density of 2 × 10^4^ cells/cm^2^ onto cell culture plates with StemFit medium (Ajinomoto) supplemented with 0.25 µg/cm^2^ iMatrix-511 (Nippi, Tokyo, Japan), laminin-511 E8 fragment, 10 µM Y27632 (WAKO), and P/S (Thermo Fisher Scientific). The culture medium was replaced the next day with StemFit medium (Ajinomoto) supplemented with P/S (Thermo Fisher Scientific). After 4 days of incubation, the culture medium was changed daily. hiPSCs at 95% confluency were passaged in an uncoated manner using iMatrix-511 (Nippi). Then, each hiPSC line was maintained as a feeder-free culture on iMatrix-511 (Nippi).

For sequencing analysis of *ZIP13-exon2* mRNA of EDSSPD3-iPSCs, total RNA was extracted from lysates of H1 and H2 or EDSSPD3-P1 and -P2 HDFs and iPSCs, respectively, using a RNeasy Mini Kit (Qiagen, GmbH, Germany). Total RNA was used to synthesize cDNA using SuperScript IV (Thermo Fisher Scientific) according to the manufacturer’s instructions. The synthesized cDNA was used as a template for PCR using human *ZIP13-exon2* mRNA primers (Supplementary Table S1). Each PCR amplicon was analyzed by Sanger sequencing.

### Establishment of hiPSC lines expressing tetracycline-inducible human *MyoD1 *(*hMyoD*)

*MyoD1* is the master transcriptional factor that regulates the differentiation of various cell types into myocytes through its enforced expression^25,26^. For the myogenic differentiation of hiPSCs, tetracycline-inducible *hMyoD*-expressing iPSC lines (iPSCs^*MYOD*^) were established by transduction of its gene cassette-inserted *piggyBac* (PB)-based vector as described previously^15,18,20^. Briefly, the plasmids for tetracycline-inducible *hMyoD* and *mCherry* and the neomycin resistant gene were constitutively prepared in PB vector (PB-*hMyoD*) or EF1a promoter-driven PB transposase gene in pHL vector (pHL-EF1a-hcPBase)^15,18,20^. The feeder-free maintained hiPSCs were treated with 10 µM Y27632 (WAKO) at least 2 h prior to transduction. hiPSCs were dissociated into single cells using a detachment solution, and the cells were resuspended in resuspension buffer R (Thermo Fisher Scientific). Two micrograms of PB-*hMyoD* and pHL-EF1a-hcPBase mixtures (1 µg each) were electroporated into 5 × 10^5^ cells using the Neon transfection system (Thermo Fisher Scientific). Electroporation conditions were 1200 V, 20 ms, and 2 time pulses. Transfected hiPSCs were incubated as a feeder-free culture on iMatrix-511 (Nippi). After 2 days, the culture medium was replaced with StemFit medium (Ajinomoto) supplemented with 2–4 mg/mL neomycin (Nacalai Tesque), and the cells were subjected to antibiotic selection. Each neomycin-selected hiPSC was cloned by limiting dilution. The established *hMyoD*-expressing hiPSC lines, named iPSCs^*MYOD*^, were confirmed based on the expression of *mCherry* in the presence of 1 µg/mL doxycycline (DOX; LKT Laboratories, Saint Paul, MN, USA). The obtained clones and 253G4^*MYOD*^ #35 which is a healthy-hiPSCs clone 253G4^27,28^ expressing tetracycline-inducible human *MyoD1*^18^ were maintained as a feeder-free culture on iMatrix-511 (Nippi).

### Correction of ***ZIP13***^***G64D***^ in EDSSPD3-iPSCs^***MYOD***^ by Cas9/sgRNA and single-stranded oligodeoxynucleotide (ssODN) electroporation

*ZIP13*^*G64D*^ in EDSSPD3-P1-iPSCs^*MYOD*^ was edited using homology-directed repair (HDR) combined with the CRISPR-Cas9 system and ssODNs^29^. We designed ssODN containing silent mutations at amino acid positions 65 (TCC < TCA, Ser) and 66 (CTC < CTA, Leu) to prevent Cas9 from recutting the *ZIP13-exon2* in the genome. GGG site showed the sequence of protospacer adjacent motif for Cas9 nuclease target. The day before transduction, EDSSPD3-P1-iPSCs^*MYOD*^ were treated with 10 µM Y27632 (WAKO). hiPSCs^*MYOD*^ were dissociated into single cells using a detachment solution, and the cells were resuspended in resuspension buffer R (Thermo Fisher Scientific). For ribonucleotide protein (RNP) electroporation, 2 µg TrueCut Cas9 protein v2 (Thermo Fisher Scientific) and 500 ng scaffold-modified *ZIP13*^*G64D*^_sgRNA (Thermo Fisher Scientific; Supplementary Table S2) were incubated for 5 min at 25 °C, and then 8 × 10^4^ cells in resuspension buffer R (Thermo Fisher Scientific), 3 µg Alt-R HDR-modified *ZIP13*^*G64D*^-corrected_ssODN (IDT, Newark, NJ, USA; Supplementary Table S2), and 3 µM electroporation enhancer (IDT) were added. Cas9/sgRNA complex and ssODN mixture was electroporated into iPSCs^*MYOD*^ using the Neon transfection system (Thermo Fisher Scientific). Electroporation conditions were 1050 V, 20 ms, and 2 time pulses. Transfected EDSSPD3-P1-iPSCs^*MYOD*^ were incubated as a feeder-free culture on iMatrix-511 (Nippi) with StemFit medium (Ajinomoto) supplemented with 30 µM Alt-R HDR enhancer (IDT). The culture medium was replaced daily with StemFit medium (Ajinomoto). After 3 days of incubation, iPSCs^*MYOD*^ were cloned by limiting dilution. The obtained clones were maintained as a feeder-free culture on iMatrix-511 (Nippi).

For sequencing analysis of human *ZIP13-exon2*, genomic DNA was extracted from lysates of transfected EDSSPD3-P1-iPSC^*MYOD*^ clones using Geno *Plus* Mini kit (VIOGENE, New Taipei, Taiwan). The genomic DNA was used as a template for PCR using human *ZIP13-exon2* genome primers (Supplementary Table S1). Each PCR amplicon was analyzed by Sanger sequencing. For separate sequencing analysis of individual alleles in a genome, genomic DNA of *ZIP13*^*G64D*^-corrected EDSSPD3-P1-iPSC^*MYOD*^ clone (repaired clone; RC) 1–3 was used as a template for PCR using human *ZIP13-exon2* genome primers for In-Fusion_pGEM (Supplementary Table S1). Their PCR amplicons were inserted in pGEM-T easy vector (Promega, Madison, WI, USA) using In-Fusion cloning kit (Takara Bio, Siga, Japan) according to the manufacturer’s instructions. The PCR amplicon-inserted plasmids were cloned and analyzed by Sanger sequencing.

### Myogenic differentiation of iPSCs^***MYOD***^

Differentiation of EDSSPD3-iPSCs^*MYOD*^ into myocytes was induced by forced *hMyoD* expression under the control of DOX as described previously^19,30^. A scheme for myogenic differentiation protocol of feeder-free iPSCs^*MYOD*^ with DOX-inducible *hMyoD* expression is shown in Supplementary Figure S5a. Myogenic differentiation using H1-iPSCs^*MYOD*^ and EDSSPD3-P1-iPSCs^*MYOD*^, H2-iPSCs^*MYOD*^ and EDSSPD3-P2-iPSCs^*MYOD*^, or H1-iPSCs^*MYOD*^, H2-iPSCs^*MYOD*^, and 253G4^*MYOD*^ #35 were performed simultaneously, respectively. Briefly, a 24-well cell culture plate was coated with Matrigel Growth Factor Reduced Basement Membrane Matrix (Corning, Corning, NY, USA) at least 2 h prior to cell seeding. Feeder-free cultured iPSCs^*MYOD*^ were dissociated into single cells using a detachment solution, and 0.6 × 10^5^ cells/well were seeded on the Matrigel-coated 24-well plate with StemFit medium (Ajinomoto) supplemented with 10 µM Y27632 (WAKO). The culture medium was replaced the next day with primate ESC medium (ReproCELL). On Day 2, 1 µg/mL DOX (LKT Laboratories) was added to the same medium, while on Day 3 the latter was replaced with minimum essential medium Eagle, alpha modification (Nacalai Tesque) supplemented with 5% KSR (Thermo Fisher Scientific), 200 µM 2-mercaptoethanol (Thermo Fisher Scientific), and 1 µg/mL DOX (LKT Laboratories). *mCherry* expression in differentiated cells on Day 3 was observed using a fluorescence microscope (*BIOREVO* BZ-9000; Keyence, Osaka, Japan). The medium was changed on Days 4, 5, and 7. Myogenic differentiation was performed at 37 °C in the presence of 5% CO_2_ until Day 8.

#### RT-qPCR

Total RNA of myogenic differentiated C2C12 cells or iPSCs^*MYOD*^ was extracted from cell lysates using a Sepazol (Nacalai Tesque) or RNeasy Mini kit (Qiagen) and was used to synthesize cDNA with the PrimeScript RT Reagent Kit (Takara Bio) or SuperScript VILO (Thermo Fisher Scientific) according to the manufacturer’s instructions. The synthesized cDNA was used as a template for qPCR, which was performed using the SYBR Green real-time PCR Master Mix (TOYOBO, Osaka, Japan). The gene-specific primers used for qPCR are provided in Supplementary Table S1. PCR and data analyses were performed using a QuantStudio3 Applied Biosystems instrument (Thermo Fisher Scientific) or an Applied Biosystems StepOnePlus real-time PCR system (Thermo Fisher Scientific). The expression of each mRNA was normalized to that of mouse *β-actin* or human glyceraldehyde 3-phosphate dehydrogenase genes (*GAPDH*).

#### Western blot (WB) analysis

Cells were lysed with RIPA buffer (Nacalai Tesque) supplemented with 0.1% bromophenol blue and 10% β-mercaptoethanol and denatured for 5 min. Cell lysates were then loaded onto a 6–10% polyacrylamide gel and subjected to SDS–PAGE. The separated proteins were transferred onto a polyvinylidene fluoride microporous membrane (Millipore, Burlington, MA, USA), and the membrane was blocked with 5% skim milk in tris-buffered saline with Tween 20. Respective primary and secondary antibodies (Supplementary Table S3) were used to detect specific proteins. The signals were detected using Immobilon Western Chemiluminescent Horseradish Peroxidase Substrate (Millipore). Signal intensities were measured using ImageJ software (National Institutes of Health, Bethesda, MD, USA), and target protein levels were normalized to that of β-ACTIN and relative protein levels were calculated. Uncropped images of the western blotting membranes are shown in Supplementary Figures S10–S14.

#### Alkaline phosphatase (ALP) staining

hiPSC colonies were fixed using 4% paraformaldehyde in DPBS(−) (Nacalai Tesque) for 10 min at 25 °C. After washing with water, and ALP staining was performed using an Alkaline Phosphatase Kit (Sigma-Aldrich) according to the manufacturer's instructions. ALP-positive hiPSC colonies stained red, and the stained colonies were photographed under phase contrast using a fluorescence microscope (*BIOREVO* BZ-9000; Keyence) without a fluorescent laser.

#### Immunofluorescence (IF) staining

Cells were fixed using 4% paraformaldehyde for 30 min at 4 °C and subsequently permeabilized by the addition of 0.3% Triton X-100 for 20 min at 25 °C. The cells were then washed with DPBS(−) and blocked using a stain buffer supplemented with FBS (BD Biosciences, Franklin Lakes, NJ, USA). Respective primary and secondary antibodies (Supplementary Table S3) were used to detect specific proteins. Cell nuclei were then stained using diamidino-2-phenylindole (DAPI; Thermo Fisher Scientific). Wells were photographed using a fluorescence microscope (*BIOREVO* BZ-9000; Keyence).

#### Flow cytometry (FC)

Feeder-free maintained hiPSCs were dissociated into single cells using a detachment solution for 10 min at 25 °C, and then suspended in a stain buffer with FBS (BD Biosciences). The cells were stained with primary antibodies for 2 h at 4 °C, followed by a secondary antibody for 1 h at 4 °C. The antibodies used for FC analysis are listed in Supplementary Table S3. Dead cells were excluded based on 7-amino-actinomycin D (BioLegend, San Diego, CA, USA) staining. Finally, the stained cells were analyzed using the BD FACSMelody cell sorter (BD Biosciences), and the data were analyzed using FlowJo software (Tree Star, Ashland, OR, USA).

#### Statistical analysis

All results are presented as mean ± standard error of the mean. The statistical significance between two groups was analyzed by Student’s *t*-test, whereas that between more than two groups was analyzed by one-way analysis of variance with the post-hoc Tukey’s test. Results were considered statistically significant at *P* < 0.05.

## Results

### Role of ZIP13 in the normal myogenic differentiation of C2C12 cells

To clarify the involvement of ZIP13 in skeletal muscle differentiation in vitro, we used C2C12 cells, which are commonly used for studying myotube differentiation (Fig. 1a). We found that *Zip13* mRNA expression was upregulated by myogenic stimulation (Fig. 1b), which occurred after upregulation of MRFs such as *MyoD* and *Myogenin* (Fig. 1c and d; Supplementary Fig. S1). Silencing of *Zip13* resulted in the suppression of myogenic differentiation (Fig. 1e–k; Supplementary Fig. S1–S3). These results suggest the possible involvement of ZIP13 in myogenic differentiation in C2C12 cells.

### Generation of iPSCs from HDFs of patients with EDSSPD3

**Figure 2:**
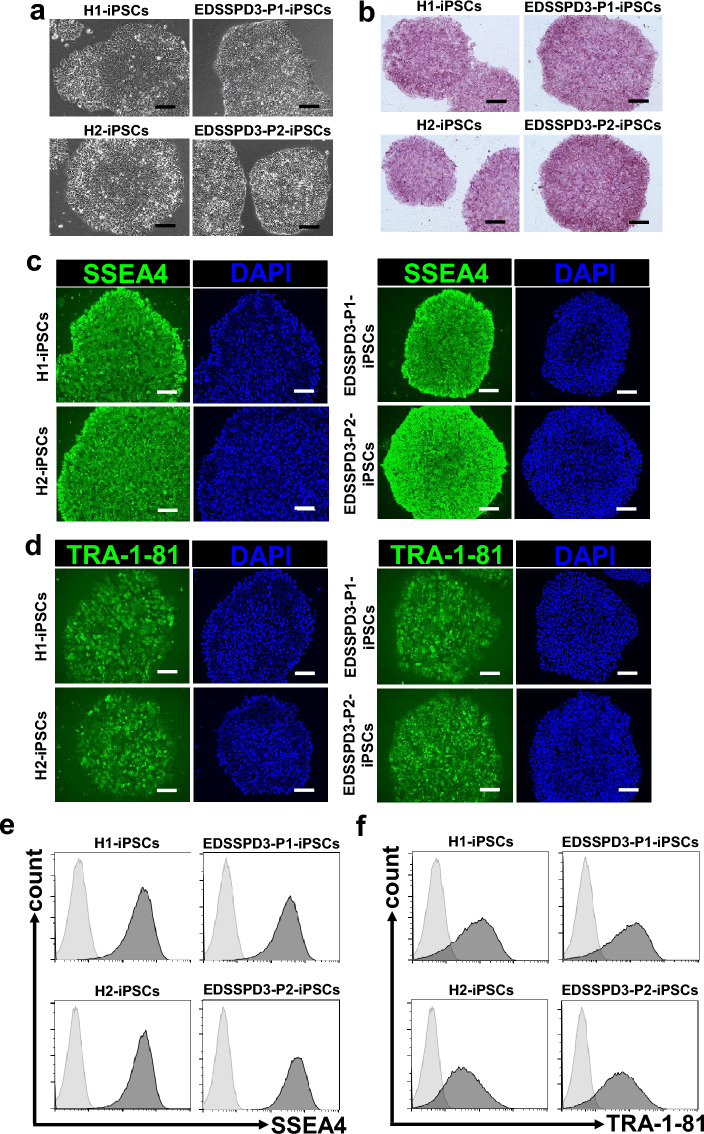
Generation and characterization of undifferentiated induced pluripotent stem cells (iPSCs) from healthy controls and patients with Ehlers–Danlos syndrome spondylodysplastic type 3 (EDSSPD3) harboring *ZIP13*^*G64D*^ mutation. iPSCs derived from healthy controls (H1 and H2) and patients with EDSSPD3 harboring *ZIP13*^*G64D*^ mutation (EDSSPD3-P1 and EDSSPD3-P2) were generated from dermal fibroblasts in an integration-free manner using episomal plasmid vectors. Each hiPSC line was maintained as a feeder-free culture on iMatrix-511. Undifferentiated H1, H2, EDSSPD3-P1, and EDSSPD3-P2 iPSC colonies were assessed by morphological examination (**a**) or alkaline phosphatase staining (**b**). Scale bar, 100 µm. (**c**,**d**) The surface expression of undifferentiated stem cell markers, SSEA4 (c) and TRA-1-81 (**d**) (green), in H1, H2, EDSSPD3-P1, and EDSSPD3-P2 iPSC colonies were analyzed by immunofluorescence (IF) staining. Cell nuclei were stained using DAPI (blue). Scale bar, 100 µm. (**e**,**f**) Flow cytometric analysis of SSEA4 and TRA-1-81 in H1, H2, EDSSPD3-P1, and EDSSPD3-P2. Mouse IgG3 antibody was used as an isotype control. Flow cytometric histograms were overlayed with data of SSEA4 (**e**) or TRA-1-81 (**f**) and mouse IgG3 isotype control. Dead cells were excluded using 7-amino-actinomycin D staining. Data are representative of three independent experiments.

To further evaluate the indispensable role of ZIP13 in human myogenic differentiation, we generated iPSCs from HDFs of patients with EDSSPD3. First, we generated iPSCs from HDFs of H1, H2, EDSSPD3-P1, and EDSSPD3-P2 by transfecting HDFs with episomal plasmid vectors. The morphological characteristics and ALP activity of undifferentiated EDSSPD3-iPSCs were equivalent to those of control iPSCs (Fig. 2a,b), both of which exhibited the same features of previously reported iPSCs^27^. We further confirmed the expression of undifferentiated-cell surface markers SSEA4 (Fig. 2c,e) and TRA-1-81 (Fig. 2d,f) in all hiPSCs and confirmed that all EDSSPD3-iPSCs harbored the original mutation *ZIP13*^*G64D*^ (Supplementary Fig. S4).

Next, we generated iPSCs^*MYOD*^ and differentiated them into myocytes under the control of DOX (Supplementary Fig. S5). The expression of exogenous genes in these cells was monitored through the expression of *mCherry* and *Exo-MYOD*, which showed equivalent expression, suggesting that iPSCs^*MYOD*^ exhibited dosage-dependent responses similar to DOX treatment (Supplementary Fig. S5b–g), whose expression were gradually downregulated during DOX-induced myogenic stimulation (Supplementary Fig. S5d–g) as previously reported^30,31^. The expression patterns of *mCherry* and *Exo-MYOD* mRNA indicate DOX-induced myogenic stimulation occurred in iPSCs^*MYOD*^, suggesting that we established iPSC^*MYOD*^ lines of healthy controls (H1-iPSCs^*MYOD*^ and H2-iPSCs^*MYOD*^) and patients with EDSSPD3 (EDSSPD3-P1-iPSCs^*MYOD*^ and EDSSPD3-P2-iPSCs^*MYOD*^).

### Characterization of myocytes differentiated from EDSSPD3-iPSCs

To assess whether the pathogenic *ZIP13*^*G64D*^ mutation has an impact on myogenic processes, we induced myogenic differentiation in iPSCs^*MYOD*^ through DOX-inducible *hMyoD* overexpression (Supplementary Fig. S5). First, to examine variabilities on myogenic differentiation between healthy controls, all the healthy control iPSCs^*MYOD*^ clones, H1-iPSCs^*MYOD*^, H2-iPSCs^*MYOD*^, and 253G4^*MYOD*^ #35 were simultaneously differentiated into myocytes with the similar trends by the same dose of DOX treatment (Supplementary Fig. S6). The expression of exogenous genes in these cells were monitored through the expression of *mCherry* and *Exo-MYOD* (Supplementary Fig. S6a, c, and d). They exhibited comparable spindle shape and expression level of myosin heavy chain (MYH) protein as noted through IF staining (Supplementary Fig. S6b). They enhanced the expression of *hZIP13* during myogenic differentiation (Supplementary Fig. S6e), and the same was also true for the expression changes of myogenic differentiation marker mRNAs, such as human endogenous-*MYOD* (*hEndo-MYOD*) and *hMYOGENIN* (Supplementary Fig. S6f. and g), and their proteins (Supplementary Fig. S6h–j). Considering that 253G4^*MYOD*^ #35 is the representative healthy control hiPSCs clone, these results suggest that H1-iPSCs^*MYOD*^ and H2-iPSCs^*MYOD*^ that we established could be used as healthy controls for experiments of myogenic differentiation.

**Figure 3:**
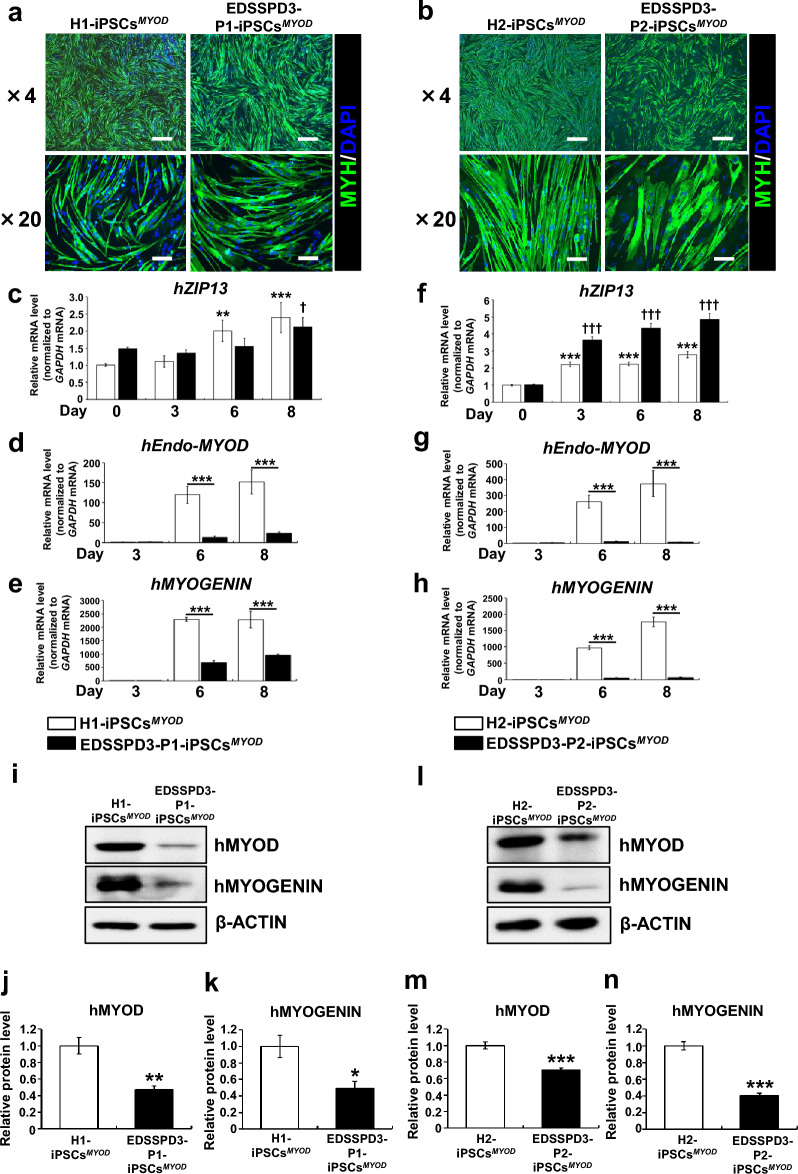
Characterization of myocytes differentiated from EDSSPD3-iPSCs^*MYOD*^ harboring the *ZIP13*^*G64D*^ mutation. Differentiations of H1-iPSCs^*MYOD*^ and H2-iPSCs^*MYOD*^ or EDSSPD3-P1-iPSCs^*MYOD*^ and EDSSPD3-P2-iPSCs^*MYOD*^ into myocytes were induced by *hMyoD* overexpression under the control of DOX as depicted in Supplementary Figure S5a. Myogenic differentiation using H1-iPSCs^*MYOD*^ and EDSSPD3-P1-iPSCs^*MYOD*^ or H2-iPSCs^*MYOD*^ and EDSSPD3-P2-iPSCs^*MYOD*^ was performed simultaneously, respectively. (**a**,**b**) IF staining of MYH in differentiated iPSCs^MYOD^ from H1-iPSCs^*MYOD*^ and EDSSPD3-P1-iPSCs^*MYOD*^ (**a**) or H2-iPSCs^*MYOD*^ and EDSSPD3-P2-iPSCs^*MYOD*^ (**b**). The images of MYH—(green) and DAPI-stained (blue) differentiated cells as observed on Day 8. Scale bar, 500 µm and 100 µm for × 4 and × 20 magnified images, respectively. (**c**–**h**) Relative mRNA expression analysis for human ZIP13 (*hZIP13*) gene and myogenic differentiation markers in differentiated H1-iPSCs^*MYOD*^ and EDSSPD3-P1-iPSCs^*MYOD*^ (**c**–**e**) or H2-iPSCs^*MYOD*^ and EDSSPD3-P2-iPSCs^*MYOD*^ (**f**–**h**). mRNA expression of *hZIP13* (**c**,**f**) (n = 9) was determined by RT-qPCR on Days 0, 3, 6, and 8, and expressed in relation to the levels in healthy-iPSCs^*MYOD*^ on Day 0 (set as 1). mRNA expressions of myogenic differentiation markers *hEndo-MYOD* (**d**,**g**) and *hMYOGENIN* (**e**,**h**) (n = 9 each) were determined by RT-qPCR on Days 3, 6, and 8, and expressed in relation to the levels in healthy-iPSCs^*MYOD*^ on Day 3 (set as 1). mRNA expression was normalized to that of human *GAPDH*. Data represent the mean ± SEM and are representative of three independent experiments. **P < 0.01 and ***P < 0.001 versus healthy-iPSCs^*MYOD*^ on Days 0 or 3. ^†^*P* < 0.05 and ^†††^*P* < 0.001 versus EDSSPD3-iPSCs^*MYOD*^ on Days 0 or 3. (**i**–**n**) Relative protein expression analysis for myogenic differentiation markers in differentiated H1-iPSCs^*MYOD*^ and EDSSPD3-P1-iPSCs^*MYOD*^ (**i**–**k**) and H2-iPSCs^*MYOD*^ and EDSSPD3-P2-iPSCs^*MYOD*^ (**l**–**n**). Protein levels of hMYOD and hMYOGENIN in cell lysates were analyzed by western blotting on Day 8 (**i**,**l**). β-ACTIN was used as an internal control. Signal intensities were measured using ImageJ software. Protein levels of hMYOD (**j**,**m**) and hMYOGENIN (**k**,**n**) (n = 5 each) were normalized to that of β-ACTIN and were expressed relative to the levels in healthy-iPSCs^*MYOD*^ on Day 8 (set as 1). Data represent the mean ± SEM and are representative of five independent experiments. *P < 0.05, **P < 0.01, and ***P < 0.001 versus healthy-iPSCs^*MYOD*^.

There were few apparent differences between the cells differentiated from patient-iPSCs and control iPSCs; both exhibited comparable spindle shape and expressed MYH protein (Fig. 3a,b). However, WB analysis revealed that MYH protein levels were lower in cells differentiated from EDSSPD3-P2-iPSCs^*MYOD*^ than in those differentiated from control iPSCs (Supplementary Fig. S7r and s). Next, we analyzed mRNA and protein expressions of myogenesis-related molecules. The relative mRNA expression of normal *hZIP13* or pathogenic *hZIP13*^*G64D*^ was significantly upregulated during DOX-induced myogenic differentiation in healthy controls iPSCs^*MYOD*^ or in EDSSPD3-iPSCs^*MYOD*^, respectively (Fig. 3c,f). Further, we assessed the expression of myogenic differentiation markers, and found that the mRNA and protein expression of hEndo-MYOD (Fig. 3d and g, i and l, j and m) and hMYOGENIN (Fig. 3e and h, i and l, k and n) were significantly downregulated in EDSSPD3-iPSCs^*MYOD*^. We also clarified the relative mRNA expressions of *hEndo-MYOD* and *hMYOGENIN* in myocytes differentiated from three healthy controls iPSCs^*MYOD*^ and two EDSSPD3-iPSCs^*MYOD*^ by using raw data of Fig. 3d, e, g, and h and Supplementary Figure S6f. and g, which showed that the mRNA expression levels of *hEndo-MYOD* and *hMYOGENIN* in patients were significantly decreased as compared with those of healthy controls when the data were averaged (Supplementary Fig. S8), thereby suggesting that the impairment of myogenic development in EDSSPD3-iPSCs were likely due to the biological failure by loss of function of ZIP13, rather than random effects by such as technical or environmental issues. Therefore, these results indicate that *ZIP13*^*G64D*^ mutation may be involved in the possible impairment of myogenic differentiation of iPSCs.

To investigate whether loss of ZIP13 effects on intracellular zinc levels in these cells, we assessed the mRNA expression of human metallothionein 1H (*hMT1H*), which is well-correlated with intracellular zinc levels^32^. We also analyzed the mRNA expression of skeletal muscle factors, *hMYH*, human glucose transporter type 4 (*hGLUT4*), and human creatine kinase, M-type (*hCK-M*), as well as myogenic precursor factors, *hMYF5*, human paired box 3 (*hPAX3*), and *hPAX7* (Supplementary Fig. S7). We found that the expression of *hMT1H* in the iPSCs of both patients on Day 0 was significantly higher than that of healthy controls, and its expression in iPSCs from healthy controls and patients on Day 0 was significantly higher than those on Days 3 to 8 (Supplementary Fig. S7a and h), indicating that the upregulation of *hMT1H* in patient iPSCs might reflect a dysregulation of intracellular zinc status by the *ZIP13*^*G64D*^ mutation. On the other hand, *hMT1H* expression in EDSSPD3-P1-iPSCs^*MYOD*^ on Day 3 was lower than that in H1-iPSCs ^*MYOD*^ (Supplementary Fig. S7a), whereas its expression in EDSSPD3-P2-iPSCs^*MYOD*^ on Days 3 was higher than that in H2-iPSCs^*MYOD*^ (Supplementary Fig. S7h). As the expression level of *hMT1H* after Day 3 became much lower than that on Day 0, it is difficult to judge whether such minor differences in *hMT1H* expression after Day 3 have any biological relevance.

In addition, we found that the *hCK-M* and *hPAX3* were significantly downregulated in myocytes derived from EDSSPD3-iPSCs^*MYOD*^ on Day 8 (Supplementary Fig. S7d and k, f, and m), and *hMYH*, *hGLUT4*, and *hPAX7* in EDSSPD3-P1-iPSCs^*MYOD*^ and EDSSPD3-P2-iPSCs^*MYOD*^ significantly differed with respect to that in healthy controls during myogenic differentiation (Supplementary Fig. S7b and i, c and j, g and o), although it is currently unclear whether statistically significant in changes of those genes would have biological relevance at this point.

### Effect of genomic correction of ***ZIP13***^***G64D***^ mutation on myogenic differentiation

**Figure 4:**
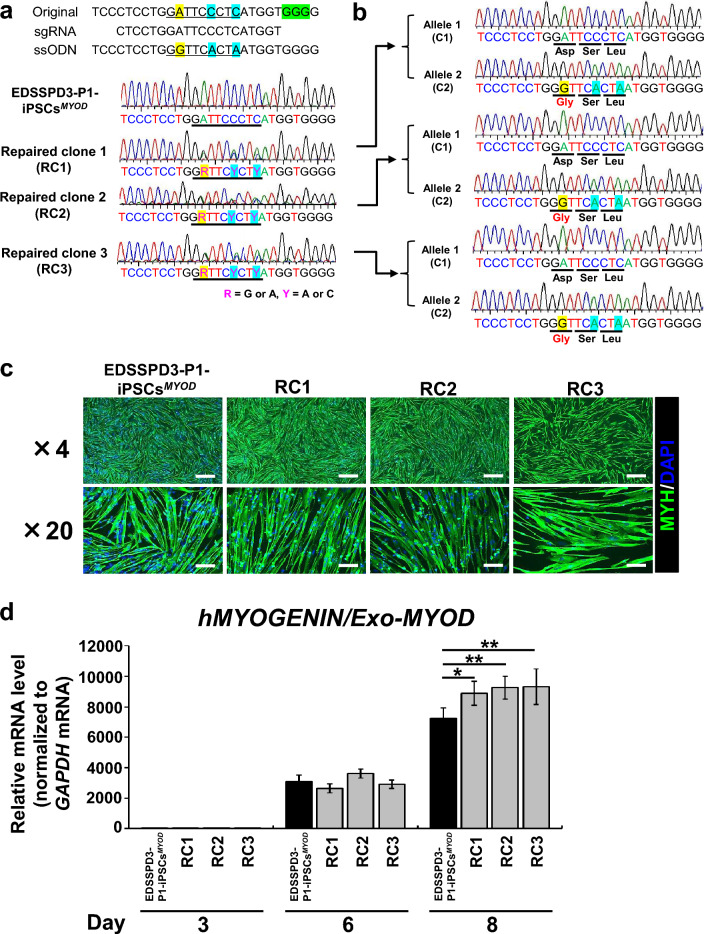
Characterization of myocytes differentiated from *ZIP13*^*G64D*^-corrected EDSSPD3-iPSCs^*MYOD*^. (**a**) We corrected *ZIP13*^*G64D*^ in EDSSPD3-P1-iPSCs^*MYOD*^ by genome editing using HDR combined with CRISPR-Cas9 system and single-stranded oligodeoxynucleotides (ssODNs). For the correction from *ZIP13*^*G64D*^ of EDSSPD3-P1-iPSCs^*MYOD*^, we used the synthesized single guide RNA (sgRNA) and ssODN (Supplementary Table S2) (**a**, upper part). After electroporation of precomplexed Cas9/sgRNA with ssODN, subclones were analyzed, and three clones of *ZIP13*^*G64D*^-corrected EDSSPD3-P1-iPSCs^*MYOD*^, designated “RC1–3”, showed allelic knockin by Sanger sequencing (R = G or A, Y = A or C) (**a**, lower part). (**b**) To sequence individual alleles separately in RC1–3, we performed In-Fusion cloning using the *ZIP13-exon2* PCR amplicons and analyzed them by Sanger sequencing. (**c**,**d**) Differentiation of EDSSPD3-P1-iPSCs^*MYOD*^ and RC1–3 into myocytes was induced by *hMyoD* overexpression under the control of DOX. A schematic depicting the protocol of myogenic differentiation of feeder-free hiPSCs^*MYOD*^ with DOX-inducible *hMyoD* expression is shown in Supplementary Figure S5a. EDSSPD3-P1-iPSCs^*MYOD*^ with homogenous alleles of *ZIP13*^*G64D*^ mutation was used as an experimental control. (**c**) IF staining of MYH in differentiated EDSSPD3-P1-iPSCs^*MYOD*^ and RC1–3. The images of MYH—(green) and DAPI-stained (blue) differentiated cells were observed on Day 8. Scale bar, 500 µm and 100 µm for × 4 and × 20 magnified images, respectively. (**d**) The relative mRNA expression of myogenic differentiation marker *hMYOGENIN* in myocytes derived from EDSSPD3-P1-iPSCs^*MYOD*^ and RC1–3 was determined by RT-qPCR, and normalized to that of human *GAPDH*. The relative expression of *hMYOGENIN* with respect to *Exo-MYOD* expression (*hMYOGENIN/Exo-MYOD*) (n = 12 each) was calculated using that of *Exo-MYOD*, as described in Supplementary Figure S9a and expressed relative to the levels in EDSSPD3-P1-iPSCs^*MYOD*^ on Day 0 (set as 1). Data represent the mean ± SEM and are representative of four independent experiments. *P < 0.05 and **P < 0.01 versus EDSSPD3-P1-iPSCs^*MYOD*^ on Day 8.

Next, to confirm the requirement of ZIP13 for myogenic differentiation, we corrected the *ZIP13*^*G64D*^ mutation in EDSSPD3-P1-iPSCs^*MYOD*^ by genome editing using HDR combined with the CRISPR-Cas9 system and ssODNs (Fig. 4a upper). *ZIP13*^*G64D*^-corrected EDSSPD3-P1-iPSCs^*MYOD*^, designated “RC1–3”, showed allelic knockin patterns as assessed by Sanger sequencing (Fig. 4a, lower). We further separately analyzed individual alleles in the genome of RC1–3 by In-Fusion cloning using the *ZIP13-exon2* PCR amplicons and found that RC1–3 had one of the corrected allele 2 (C2) (Fig. 4b). These results demonstrated that the *ZIP13*^*G64D*^ mutation in EDSSPD3-P1-iPSCs^*MYOD*^ was substituted resulting in the change of homogenous alleles to heterogeneous alleles, which are similar to the genotype of the parents of the patients with EDSSPD3 included in the present study. The parents exhibited no clinical issues because EDSSPD3 is a recessive disorder^4^. Subsequently, we induced myogenic differentiation in the corrected clones (RC1–3) using DOX treatment, and observed similar spindle shape and high MYH expression as that in the control cells (Fig. 4c). Although the relative mRNA expression of *hZIP13* in EDSSPD3-P1-iPSCs^*MYOD*^ and RC1–3 was significantly upregulated during DOX-induced myogenic differentiation (Supplementary Fig. S9b), mRNA expressions of *Exo-MYOD* and *hMT1H* were altered on Day 3 (Supplementary Fig. S9a and c). Each iPSC clone expressed *Exo-MYOD* mRNA with DOX supplementation, which was followed by myogenic differentiation^30^. The relative expression levels of *hMYOGENIN* (Fig. 4d) and hMYOGENIN protein (Supplementary Fig. S9d and e) were increased in RC1–3 on Day 8 relative to that in EDSSPD3-P1-iPSCs^*MYOD*^. These results indicate that genome editing of the *ZIP13*^*G64D*^ mutation resulted in normalized expression of myogenic differentiation markers, so that ZIP13 is possibly involved in the regulation of myogenic differentiation.

## Discussion

In the present study, using pathogenic *ZIP13*^*G64D*^ mutation-harboring iPSCs established from patients with EDSSPD3, we demonstrated that ZIP13 is possibly involved in myogenic differentiation.

Skeletal muscles accumulate the largest proportion of total zinc in body, which is about 60% of the total levels in the body^33,34^; however, physiological roles of zinc transporters in skeletal muscle are not fully understood. Patients with EDSSPD3 suffer from muscular hypotonia or myopathy, implicating the role of ZIP13 in skeletal muscles^4,6,9^. In the present study, we found that *Zip13* was upregulated in mouse C2C12 cells and human iPSCs during myogenic differentiation along with myogenic differentiation markers *MyoD* and *Myogenin.* Additionally, *Zip13* silencing suppressed myogenic differentiation. The same was also observed with EDSSPD3-iPSCs, which suggests that ZIP13 is involved in myogenic differentiation. However, we could not exclude the involvement of other factors such as culture conditions and passage numbers, which may have affected myogenesis along with the loss of ZIP13 in our experimental conditions. Intriguingly, another ZIP family member, ZIP14, reportedly acts on muscle homeostasis in a manner different to ZIP13^35^. ZIP14 was reported to be upregulated in skeletal muscles of mice and patients with metastatic cancers, while ZIP14-mediated zinc uptake in muscle progenitor cells and differentiated myocytes repressed the expression of MyoD and MYH, respectively, promoting muscle wasting in cancer cachexia^35^. These reports together with the present findings suggest that a functional balance between ZIP family members is required for muscle homeostasis, and that ZIP13 may play a role in the early stages of myogenesis.

How does ZIP13 regulate molecular events during myogenic differentiation? ZIP13 is reportedly involved in BMP/TGF-β-mediated signaling pathways and is responsible for various phenotypes and symptoms in *Zip13*-KO mice and patients with EDSSPD3 harboring *ZIP13*^*G64D*^ mutation^4,8^. Interestingly, *Zip13*-KO mice and patients with EDSSPD3 exhibit reduced white fat mass^4^. This may be because *Zip13*-KO mice showed accelerated adipocyte browning in association with increased protein levels of CCAAT/Enhancer Binding Protein β (C/EBP-β), suggesting an important role of ZIP13 in suppressing adipocyte browning through the downregulation of C/EBP-β protein levels^36^. Several studies have reported that both BMP signaling and C/EBP-β play important roles in myogenic differentiation. BMP signaling maintains the proliferative status of muscle progenitors and increases muscle mass^37,38^, while C/EBP-β plays essential roles in repressing myogenesis^39,40^. Notably, C/EBP-β is rapidly degraded upon activation by the ubiquitin–proteasome system to facilitate myogenic differentiation^39^. Consistently, C/EBP-β overexpression in myoblasts inhibits their differentiation, whereas loss of its expression promotes myogenic differentiation and myofiber development^41^. C/EBP-β expression also inhibits the transcription of *MyoD* and *Myogenin*^40,41^. In the present study, we demonstrated that loss of ZIP13 attenuated the expression of myogenic differentiation markers MyoD and Myogenin in C2C12 cells and EDSSPD3-iPSCs. Therefore, the balance between impaired BMP signaling and C/EBP-β-mediated cascades can potentially be impaired by loss of ZIP13, which may in turn result in abnormal myogenic differentiation.

To further clarify the role of ZIP13 in early skeletal muscle development, especially in humans, we derived iPSCs from HDFs of patients with EDSSPD3 harboring *ZIP13*^*G64D*^ mutation because using a patient-derived disease cell model is beneficial for elucidating the underlying pathogenetic mechanisms and has potential applications in regenerative medicine^14^. In vitro disease models using patient-derived iPSCs can also be used for high-throughput screening of drugs. In fact, in vitro models of various muscle-related diseases, such as DMD^15–17^, MM^18,19^, and Pompe disease^20^, have been used for elucidating their pathogenetic mechanisms and for drug screening. Herein, we generated iPSCs from patients with EDSSPD3 harboring *ZIP13*^*G64D*^ mutation, which were further differentiated into myoblasts by *hMyoD* overexpression. We showed that these myocytes exhibited attenuated expression of myogenic differentiation markers as compared to that in healthy controls. Previous studies have reported that myopathies may impair myogenesis via the expression of myogenic differentiation markers such as MyoD^11,42–44^ and Myogenin^12,45–47^. Taken together, these results indicate that myopathies caused by loss of ZIP13 function may initiate attenuation of the expression of myogenic differentiation markers in EDSSPD3-iPSC-derived myocytes.

To precisely elucidate the phenotypic abnormalities of EDSSPD3-iPSCs-derived myocytes and to further confirm the requirement of ZIP13 for myogenic differentiation, we performed genome editing using ssODN-mediated HDR combined with CRISPR-Cas9 system to rectify the *ZIP13*^*G64D*^ mutation. This genome editing system is widely utilized for precise genome editing at a desired locus^29^ and has been applied previously to study muscle diseases, such as DMD^48^ or MM^29^. Herein, we showed that genomic editing of the *ZIP13*^*G64D*^ mutation in EDSSPD3-iPSCs^*MYOD*^ recovered the expression of myogenic differentiation marker Myogenin in myocytes differentiated from EDSSPD3-iPSCs^*MYOD*^. These findings indicate that ZIP13 is indispensably involved in the early stages of myogenic differentiation.

It has been established that the alternation of MRFs, such as MyoD and Myogenin impacts skeletal development and/or functions^49,50^, since they are integral to the development of normal skeletal muscle through regulation of proliferation and myogenic fusion during development^51^. Indeed, Myogenin is necessary for both early muscle development^45,46^ and for myofiber growth in adults^12^. In the future, genetic murine models of the disease such as *Zip13*-deficient mice and muscle specific *Zip13*-conditional knock out mice should be employed to clearly elucidate the mechanisms underlying the pathophysiological abnormalities observed in patients with EDSSPD3. In this study, we found that the attenuated expression of myogenic differentiation markers similarly occurred in myocytes differentiated from the iPSCs of two patients with EDSSPD3 harboring the *ZIP13*^*G64D*^ mutation. However, variations in the expression levels of several muscle-associated genes were observed between the two patients (Supplementary Fig. S7), which makes it difficult to conclude whether such statistically significant data indicate any biological relevance that could define and characterize these cells. Thus, further research is necessary to fully clarify the role of ZIP13 in myogenesis.

**Figure 5:**
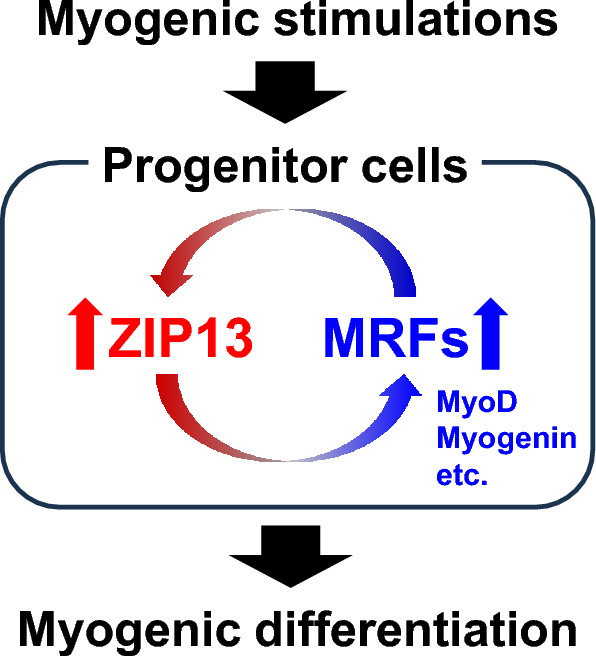
ZIP13 is involved in normal myogenic differentiation. ZIP13 is upregulated under myogenic stimulation, most likely through myogenic regulatory factors (MRFs) including MyoD and Myogenin. In addition, ZIP13 and MRFs, namely MyoD and Myogenin, are required for induction with each other, implying that ZIP13 might have a role to amplify the expression loop of MRFs leading the accurate myogenic differentiation.

In conclusion, we demonstrated that ZIP13 is involved in myogenic differentiation (Fig. 5). Our findings suggest that ZIP13 may be a novel MRF that regulates the expression of MyoD and Myogenin to facilitate accurate myogenesis. Recently, we reported the possible involvement of ZIP13 in the control of cardiovascular system^52^. Although further research is necessary to clarify the role of ZIP13 in the physiological functions of affected tissues including the heart and skeletal muscles, the patient-derived iPSCs established in the present study could be useful not only to uncover the mechanisms underlying ZIP13-mediated biological functions, but can also facilitate regenerative studies and pharmaceutical applications for the treatment of EDSSPD3.

### Supplementary Information

Supplementary Information.

## Data Availability

All data generated or analyzed during this study are included in this published article and its Supplementary Information files. Raw sequencing data described in Supplementary Figure S4 and Fig. 4a and b were deposited in DNA Data Bank of Japan (DDBJ) that belongs to the International Nucleotide Sequence Database Collection (INSDC) under accession numbers LC741039–42 and LC741043–46 and LC741047–50 and LC74104351–56, respectively. The datasets used and/or analyzed during the current study are available from the corresponding author on reasonable request.
