# Supplementary material for: Possible involvement of zinc transporter ZIP13 in myogenic differentiation
Source: Sci Rep. 2024 Apr 12;14:8052. doi: 10.1038/s41598-024-56912-7 (PMC11014994; doi:10.1038/s41598-024-56912-7)
Supplement: Supplementary file 1 — Supplementary Information. [file 41598_2024_56912_MOESM1_ESM.pdf]

Supplementary Information

Possible involvement of zinc transporter ZIP13 in myogenic differentiation

Masaki Shoji[1,†,*], Takuto Ohashi[2,†], Saki Nagase[1], Haato Yuri[1], Kenta Ichihashi[1], Teruhisa Takagishi[2], Yuji Nagata[2], Yuki Nomura[2], Ayako Fukunaka[3], Sae, Kenjou[2], Hatsuna Miyake[2], Takafumi Hara[2], Emi Yoshigai[2], Yoshio Fujitani[3], Hidetoshi Sakurai[4], Heloísa G. dos Santos[5], Toshiyuki Fukada[2,*], and Takashi Kuzuhara[1,*]

[1] Laboratory of Biochemistry, Faculty of Pharmaceutical Sciences, Tokushima Bunri University, Tokushima, Japan.
[2] Laboratory of Molecular and Cellular Physiology, Faculty of Pharmaceutical Sciences, Tokushima Bunri University, Tokushima, Japan.
[3] Laboratory of Developmental Biology and Metabolism, Institute for Molecular and Cellular Regulation, Gunma University, Maebashi, Japan.
[4] Center for iPS Cell Research and Application (CiRA), Kyoto University, Kyoto, Japan.
[5] Serviço Genética Médica, Hospital S. Maria, Lisboa, Portugal.

[†]These authors contributed equally to this study.
[*]Corresponding authors:
Laboratory of Biochemistry, Faculty of Pharmaceutical Sciences, Tokushima Bunri University, 180 Nishihamahouji, Yamashirocho, Tokushima-city, Tokushima 770-8514, Japan. Tel.: +81 (88) 602-8477; Fax: +81 (88) 655-3051. E-mail address: masaki-shoji@ph.bunri-u.ac.jp (M.S.) and kuzuhara@ph.bunri-u.ac.jp (T.K.).
Laboratory of Molecular and Cellular Physiology, Faculty of Pharmaceutical Sciences, Tokushima Bunri University, 180 Nishihamahouji, Yamashirocho, Tokushima-city, Tokushima 770-8514, Japan. Tel.: +81 (88) 602-8593; Fax: +81 (88) 655-3051.
E-mail address: fukada@ph.bunri-u.ac.jp (T.F.).

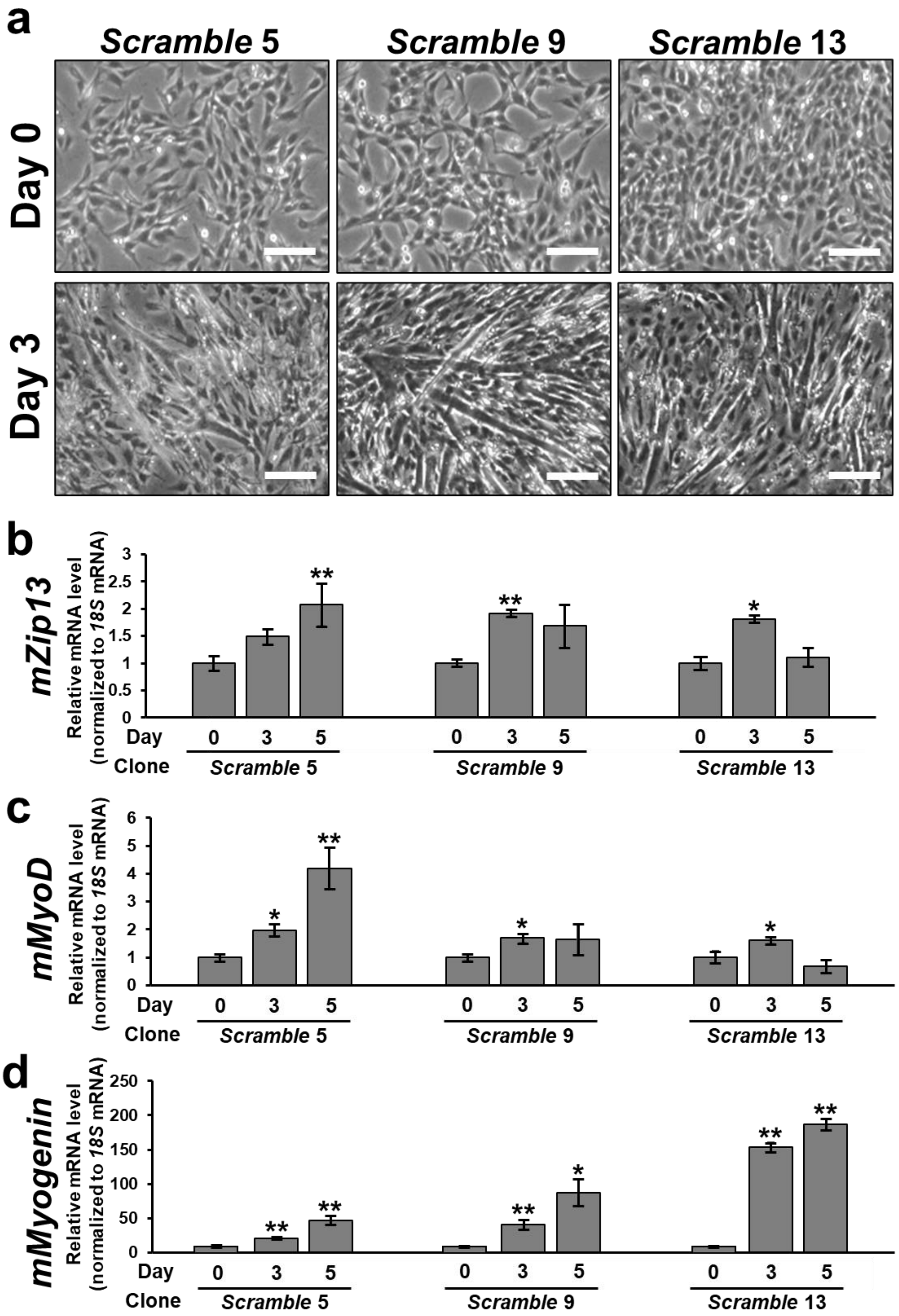

Supplementary Figure S1. Myogenic differentiation in the *Scramble* clones of C2C12 cells. Differentiations of *Scramble* clones (clone number: 5, 9, and 13) of C2C12 cells

into myocytes were induced with Dulbecco's modified eagle medium (DMEM) containing 2% horse serum (HS) as depicted in Figure 1a. (**a**) Microscopic evaluation of the morphological changes in *Scramble* clones of C2C12 cells (clone number: 5, 9, and 13) during myogenic differentiation on Days 0 and 3. Scale bars, 150 μm. (**b**–**d**) Gene expression profiles of HS-induced *mZip13* (**b**) and myogenic differentiation markers, *mMyoD* (**c**) and *mMyogenin* (**d**), (n = 3 each) analyzed by qPCR. The cells were harvested at the indicated time points. Data are presented as the mean ± standard error of mean (SEM) of three independent experiments. *P < 0.05 and **P < 0.01 relative to the data on Day 0.

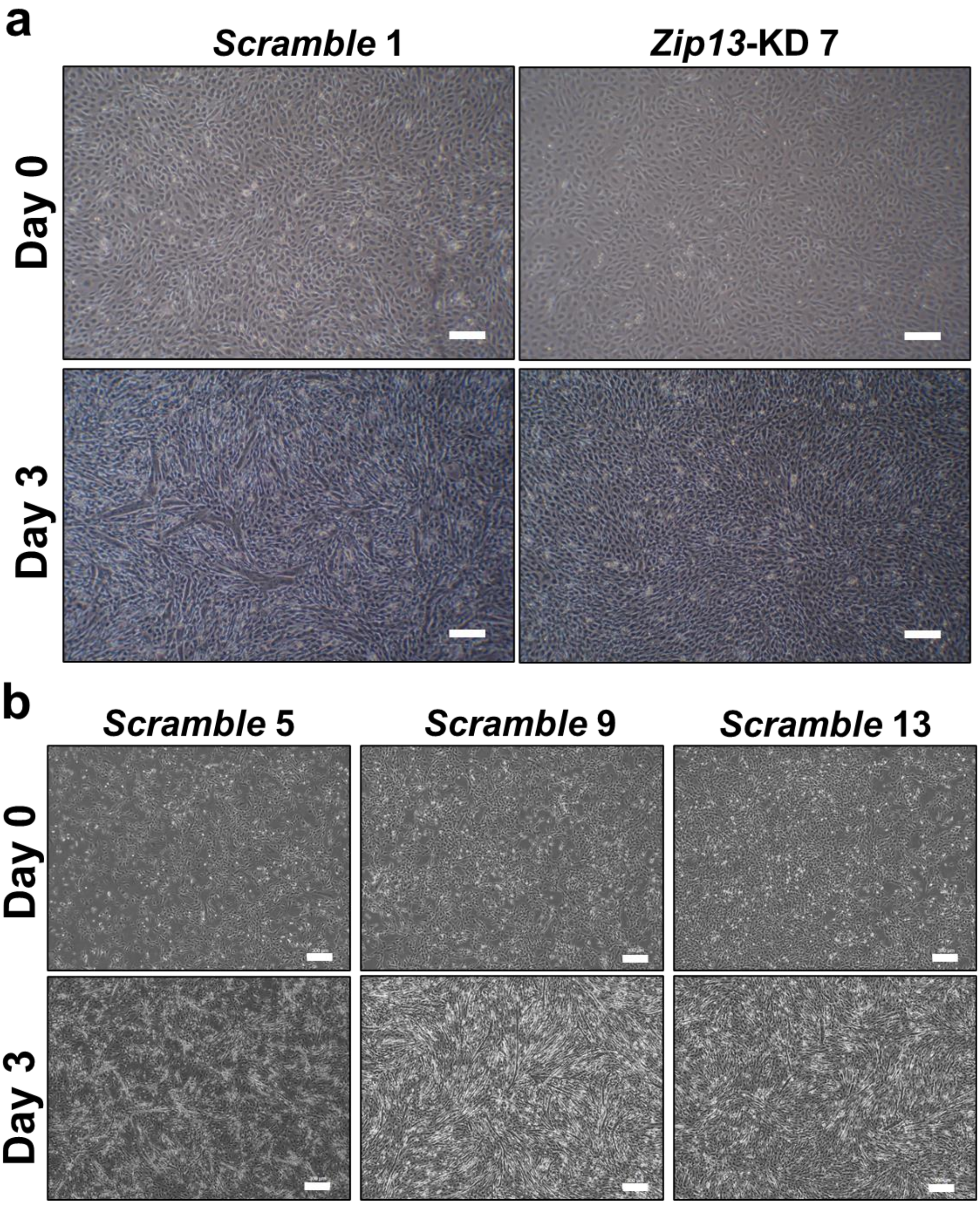

Supplementary Figure S2. Downscaled microscopic images in myogenic differentiated *Scramble* or *Zip13*-KD clones of C2C12 cells. Downscaled microscopic images of Figure 1f (**a**) and Supplementary Figure S1a (**b**) were shown. Scale bars, 300 μm.

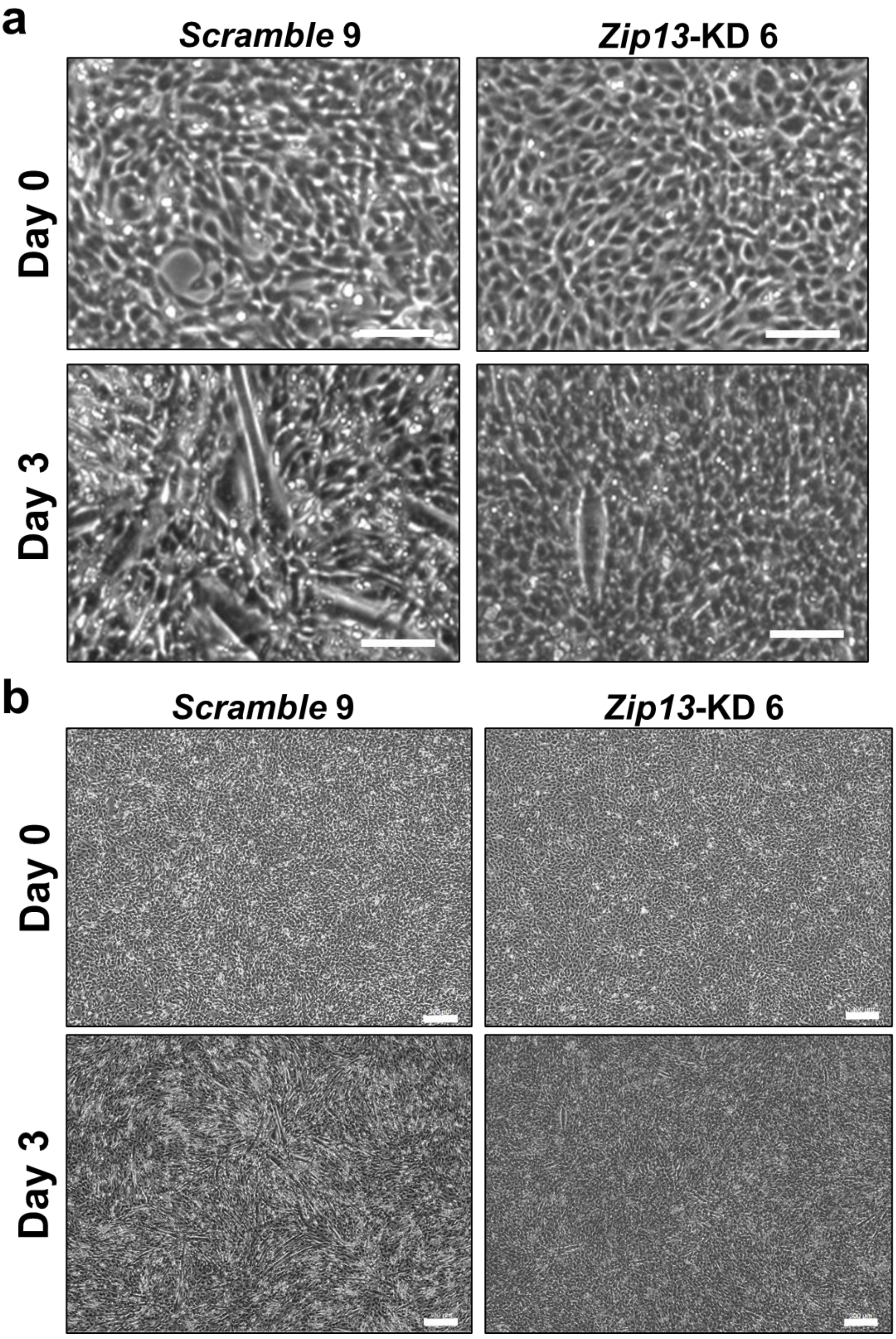

Supplementary Figure S3. Myogenic differentiation in *Scramble* 9 and *Zip13*-KD 6 of C2C12 cells. Differentiations of *Scramble* 9 and *Zip13*-KD 6 of C2C12 cells into myocytes were induced, as depicted in Figure 1a. (**a**) Microscopic images of the

morphological changes in *Scramble* 9 and *Zip13*-KD 6 of C2C12 cells at the indicated time points after myogenic differentiation stimuli with DMEM containing 2% HS. Scale bars, 150 μm. (**b**) Downscaled microscopic images of Supplementary Figure S3a were shown. Scale bars, 300 μm.

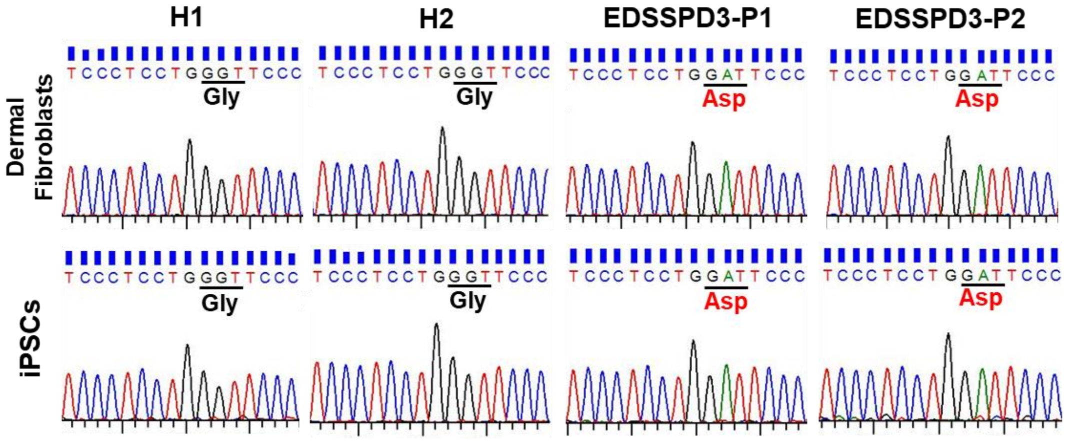

Supplementary Figure S4. Sequencing analysis of *ZIP13-exon2* mRNA from iPSCs derived from patients with EDSSPD3 harboring *ZIP13*^{G64D} mutation. Total RNA was extracted from the lysates of dermal fibroblasts and iPSCs from healthy controls (H1 and H2) or patients with EDSSPD3 harboring *ZIP13*^{G64D} mutation (EDSSPD3-P1 and EDSSPD3-P2), and used to synthesize cDNA. The synthesized cDNA was used as a template for PCR using human *ZIP13-exon2* mRNA primers. Their PCR amplicons were analyzed by Sanger sequencing. Codon GGT: Glycine (Gly), GAT: Aspartic acid (Asp).

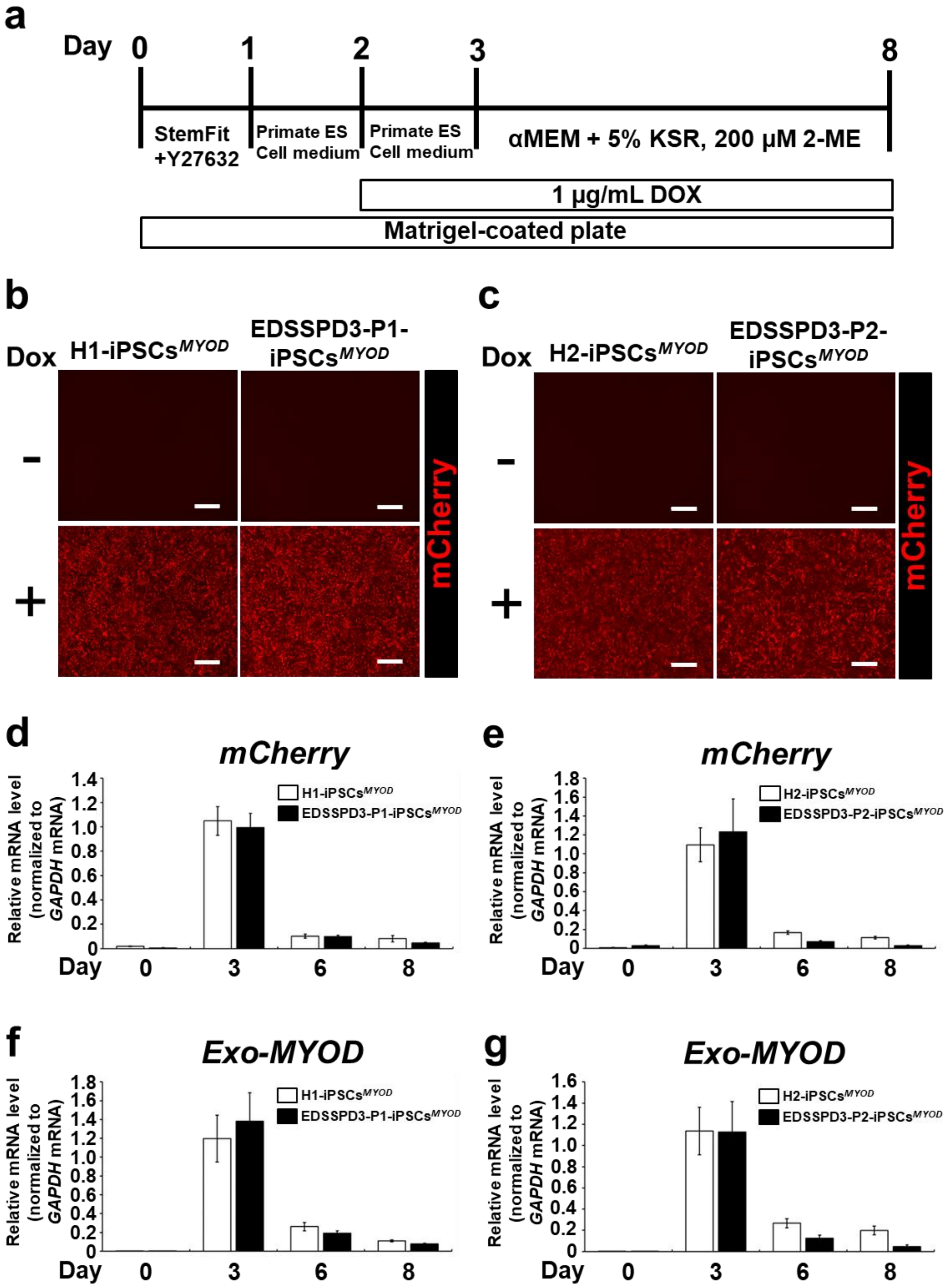

Supplementary Figure S5. Exogenous gene expressions in myogenic differentiation of feeder-free healthy- or EDSSPD3-iPSCs^{MYOD} with doxycycline (DOX)-inducible *hMyoD* expression. (**a**) A scheme for the protocol of myogenic differentiation of feeder-free hiPSCs^{MYOD} with DOX-inducible *hMyoD* expression. Feeder-free cultured

hiPSCs^{MYOD} were dissociated into single cells using a detachment solution and seeded on Matrigel-coated 24-well plate in StemFit medium supplemented with 10 µM Y27632. The culture medium was replaced the next day with primate ES cell medium. On Day 2, 1 µg/mL DOX was added to the same medium, and the latter was replaced with minimum essential medium eagle, alpha modification (αMEM) supplemented with 5% knockout serum replacement (KSR), 200 µM 2-mercaptoethanol (2-ME), and 1 µg/mL DOX on Day 3. This myogenic differentiation was performed at 37 °C in the presence of 5% CO_2 until Day 8. (**b**–**g**) Differentiation of iPSCs^{MYOD} from healthy controls (H1-iPSCs^{MYOD} and H2-iPSCs^{MYOD}) and patients with EDSSPD3 (EDSSPD3-P1-iPSCs^{MYOD} and EDSSPD3-P2-iPSCs^{MYOD}) into myocytes was induced by *hMyoD* overexpression under the control of DOX, as s according to Supplementary Figure S5a. The experiments of myogenic differentiation using H1-iPSCs^{MYOD} and EDSSPD3-P1-iPSCs^{MYOD} or H2-iPSCs^{MYOD} and EDSSPD3-P2-iPSCs^{MYOD} were performed simultaneously, respectively. (**b** and **c**) mCherry expression (red) in DOX-treated or -untreated differentiated from H1-iPSCs^{MYOD} and EDSSPD3-P1-iPSCs^{MYOD} (**b**) or H2-iPSCs^{MYOD} and EDSSPD3-P2-iPSCs^{MYOD} (**c**) was observed by fluorescence microscopy on Day 3. Scale bars, 500 µm. (**d**–**g**) The relative mRNA expressions of exogenous genes *mCherry* (**d** and **e**) or *Exo-MYOD* (**f** and **g**) in differentiated from H1-iPSCs^{MYOD} and EDSSPD3-P1-iPSCs^{MYOD} (**d** and **f**) or H2-iPSCs^{MYOD} and EDSSPD3-P2-iPSCs^{MYOD} (**e** and **g**) (n = 9 each), as determined by RT-qPCR, on Days 0, 3, 6, and 8, normalized to that of human *GAPDH* and expressed relative to the levels in healthy-iPSCs^{MYOD} on Day 3 (set as 1). Data are presented as mean ± SEM and are representative of three independent experiments.

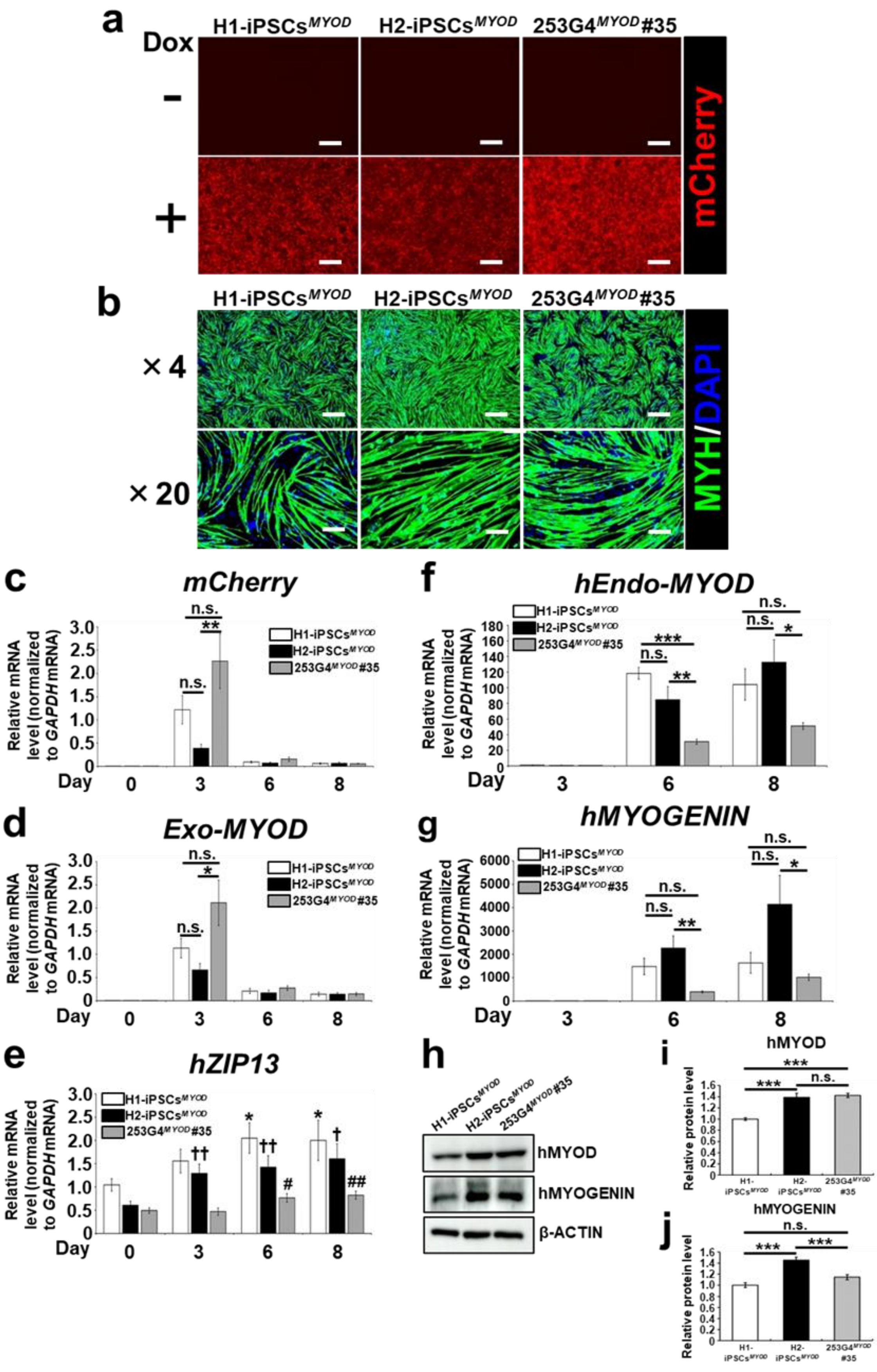

a
Dox
H1-iPSCsMYOD
H2-iPSCsMYOD
253G4MYOD#35
-
+
mCherry
b
×4
×20
MYH/DAPI
c
mCherry
Relative mRNA level (normalized to GAPDH mRNA)
Day
d
Exo-MYOD
e
hZIP13
f
hEndo-MYOD
g
hMYOGENIN
h
hMYOD
hMYOGENIN
β-ACTIN
i
hMYOD
Relative protein level
j
hMYOGENIN
n.s.

Supplementary Figure S6. Myogenic differentiation in clones of feeder-free cultured healthy-hiPSCs^{MYOD} with DOX-inducible *hMyoD* expression. Differentiation of iPSCs^{MYOD} from healthy controls (H1-iPSCs^{MYOD}, H2-iPSCs^{MYOD}, and 253G4^{MYOD}) into myocytes was induced by *hMyoD* overexpression under the control of DOX, according to Supplementary Figure S5a. The experiments of myogenic differentiation using H1-iPSCs^{MYOD}, H2-iPSCs^{MYOD}, and 253G4^{MYOD} #35 were performed simultaneously. (**a**) mCherry expressions (red) in DOX-treated or -untreated differentiated from H1-iPSCs^{MYOD}, H2-iPSCs^{MYOD}, and 253G4^{MYOD} #35 were observed by fluorescence microscopy on Day 3. Scale bars, 500 µm. (**b**) IF staining of MYH in differentiated from H1-iPSCs^{MYOD}, H2-iPSCs^{MYOD}, and 253G4^{MYOD} #35. The images of MYH- (green) and DAPI-stained (blue) differentiated cells as observed on Day 8. Scale bar, 500 µm and 100 µm for ×4 and ×20 magnified images, respectively. (**c**–**g**) The relative mRNA expressions of exogenous genes, *mCherry* (**c**) and *Exo-MYOD* (**d**), *hZIP13* (**e**), or myogenic differentiation markers, *hEndo-MYOD* (**f**) and *hMYOGENIN* (**g**), (n = 7 each) in differentiated from H1-iPSCs^{MYOD}, H2-iPSCs^{MYOD}, and 253G4^{MYOD} #35 were analyzed by RT-qPCR, on Days 0, 3, 6, and 8 or 3, 6, and 8, normalized to that of human *GAPDH* and expressed relative to the levels in H1-iPSCs^{MYOD} on Days 0 or 3 (set as 1), respectively. Data are presented as mean ± SEM and are representative of seven independent experiments. *P < 0.05, **P < 0.01, and ***P < 0.001 versus the indicated groups (**c**, **d**, **f**, and **g**). *P < 0.05 versus H1-iPSCs^{MYOD} on Day 0 (**e**). †P < 0.05 and ††P < 0.01 versus H2-iPSCs^{MYOD} on Day 0 (**e**). $^{\#}$P < 0.05 and $^{\#\#}$P < 0.01 versus 253G4^{MYOD} #35 on Day 0 (**e**). n.s.; not significant. (**h**–**j**) Relative protein expression analysis for hMYOD and hMYOGENIN in differentiated healthy controls. The protein expression levels of hMYOD and hMYOGENIN in cell lysates were analyzed by western blotting on Day 8 (**h**). β-ACTIN was used as an internal control. Signal intensities were measured using ImageJ software. The protein expression levels of hMYOD (**i**) and hMYOGENIN (**j**) (n = 32 each) were calculated after normalizing them to that of β-ACTIN and expressed relative to the levels in H1-iPSCs^{MYOD} on Day 8 (set as 1). Data represent the mean ± SEM and are representative of three independent experiments. ***P < 0.001 versus the indicated groups. n.s.; not significant.

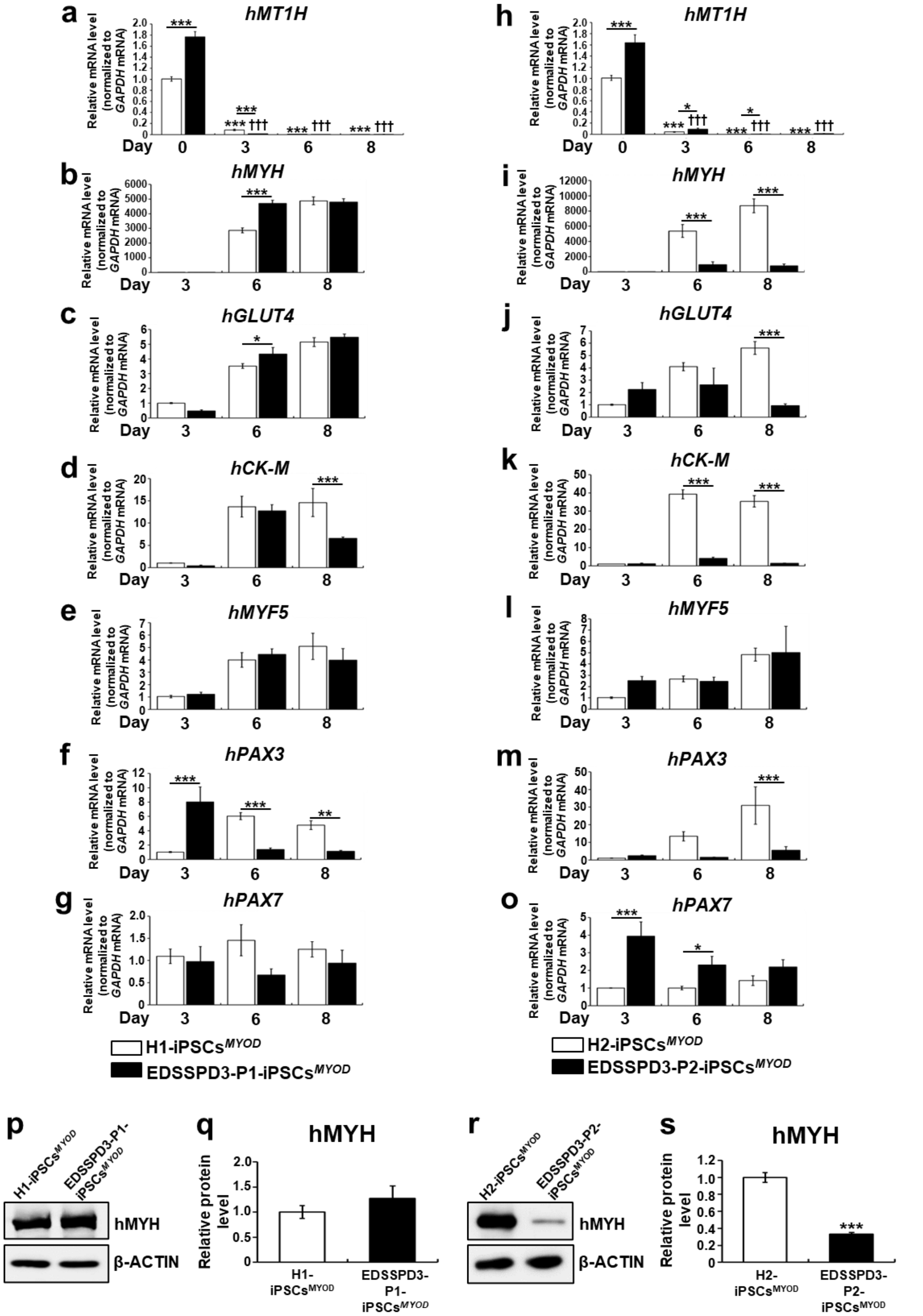

Supplementary Figure S7. The expression of skeletal muscle and myogenic precursor markers in myocytes differentiated from iPSCs^{MYOD} from patients with EDSSPD3 harboring *ZIP13*^{G64D} mutation. Differentiation of H1-iPSCs^{MYOD} and H2-

iPSCs^{MYOD} or EDSSPD3-P1-iPSCs^{MYOD} and EDSSPD3-P2-iPSCs^{MYOD} into myocytes was induced by *hMyoD* overexpression under the control of DOX according to Supplementary Figure S5a. The experiments of myogenic differentiation using H1-iPSCs^{MYOD} and EDSSPD3-P1-iPSCs^{MYOD} or H2-iPSCs^{MYOD} and EDSSPD3-P2-iPSCs^{MYOD} were performed simultaneously, respectively. (**a**–**o**) The relative mRNA expression analysis for skeletal muscle or myogenic precursor markers in differentiated H1-iPSCs^{MYOD} and EDSSPD3-P1-iPSCs^{MYOD} or H2-iPSCs^{MYOD} and EDSSPD3-P2-iPSCs^{MYOD}. The mRNA expression levels of zinc status marker, *hMT1H* (**a** and **h**) (n = 9) was determined by RT-qPCR on Days 0, 3, 6, and 8, normalized to that of human *GAPDH* and expressed relative to the levels in healthy-iPSCs^{MYOD} on Day 0 (set as 1). The mRNA expression levels of skeletal muscle markers, *hMYH* (**b** and **i**), *hGLUT4* (**c** and **j**), and *hCK-M* (**d** and **k**) and myogenic precursor markers, *hMYF* (**e** and **l**), *hPAX3* (**f** and **m**), and *hPAX7* (**g** and **o**) (n = 9 each) were determined by RT-qPCR on Days 3, 6, and 8, normalized to that of human *GAPDH* and expressed relative to the levels in healthy-iPSCs^{MYOD} on Day 3 (set as 1). Data are presented as mean ± SEM and are representative of three independent experiments. *P < 0.05, **P < 0.01, and ***P < 0.001, versus the indicated groups for healthy-iPSCs^{MYOD}. ***P < 0.001 versus healthy-iPSCs^{MYOD} on Day 0 (**a** and **h**). †††P < 0.001 versus EDSSPD3-patients-iPSCs^{MYOD} on Day 0 (**a** and **h**). (**p**–**s**) The protein expression levels of hMYH in cell lysates were analyzed by western blotting on Day 8 (**p** and **r**). β-ACTIN protein was used as an internal control. Signal intensities were measured using ImageJ software. The protein levels of hMYH (**q** and **s**) (n = 5 each) were calculated by normalizing them to that of β-ACTIN and expressed relative to the levels in healthy-iPSCs^{MYOD} on Day 8 (set as 1). Data are presented as mean ± SEM and are representative of five independent experiments. ***P < 0.001 versus healthy-iPSCs^{MYOD}.

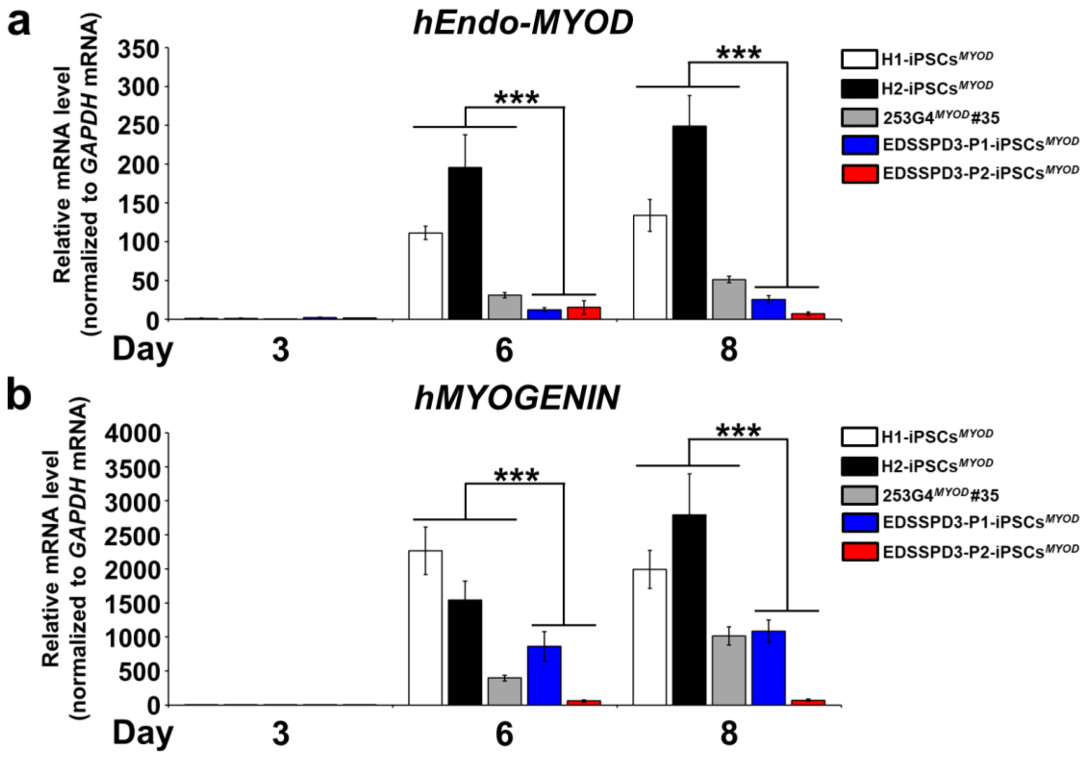

Supplementary Figure S8. The relative mRNA expressions of myogenic differentiation markers, *hMYOD* and *hMYOGENIN*, in myocytes differentiated from three healthy controls iPSCs^{MYOD} and two patients-iPSCs^{MYOD} in this study. The relative mRNA expressions of *hEndo-MYOD* (**a**) and *hMYOGENIN* (**b**) in the myocytes differentiated from three healthy controls iPSCs^{MYOD}, H1-iPSCs^{MYOD} (n = 16), H2-iPSCs^{MYOD} (n = 16), and 253G4^{MYOD} #35 (n = 7), and two patients-iPSCs^{MYOD} with EDSSPD3, EDSSPD3-P1-iPSCs^{MYOD} (n = 9) and EDSSPD3-P2-iPSCs^{MYOD} (n = 9), on Days 3, 6, and 8 were calculated with raw data in Figure 3d, e, g, and h and Supplementary Figure S6f and g, normalized to those of human *GAPDH* and expressed relative to the levels in H1-iPSCs^{MYOD} on Day 3 (set as 1). Simultaneously, we statistically analyzed the difference in these expression levels between the averaged data across three healthy controls or two patients with EDSSPD3 using Student's *t*-test. Data are presented as mean ± SEM. ***$P < 0.001$ compared to the indicated groups for healthy controls iPSCs^{MYOD}.

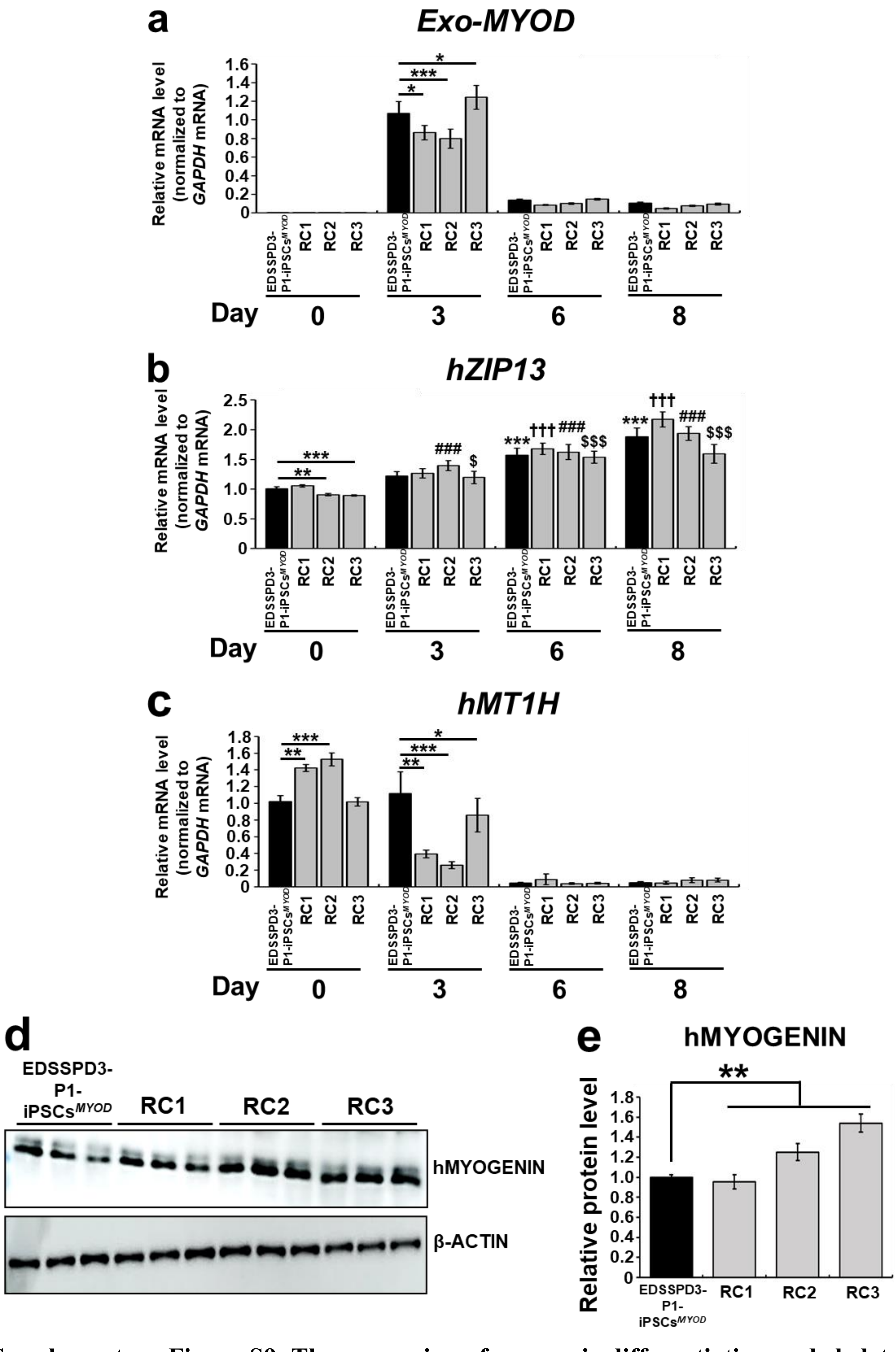

Supplementary Figure S9. The expression of myogenic differentiation and skeletal markers in myocytes differentiated from *ZIP13*^{G64D}-corrected EDSSPD3-iPSCs^{MYOD}.

The relative mRNA expressions of *Exo-MYOD* (**a**), *hZIP13* (**b**), and *hMT1H* (**c**) (n = 12 each) in the myocytes derived from EDSSPD3-P1-iPSCs^{MYOD} and its *ZIP13*^{G64D}-corrected clones (repaired clone; RC), designated RC1–3, were determined by RT-qPCR, normalized to that of human *GAPDH* and expressed relative to the levels in EDSSPD3-P1-iPSCs^{MYOD} (before *ZIP13*^{G64D}-correction) on Day 0 (set as 1). Data are presented as mean ± SEM and are representative of four independent experiments. *P < 0.05, **P < 0.01, and ***P < 0.001 versus EDSSPD3-P1-iPSCs^{MYOD} on Days 0 or 3, †††P < 0.001 versus RC1 on Day 0. $^{\#\#\#}$P < 0.001 versus RC2 on Day 0. $^{\$}$P < 0.05 and $^{\$\$\$}$P < 0.001 versus RC3 on Day 0. (**d** and **e**) Relative protein expression analysis for hMYOGENIN in differentiated EDSSPD3-P1-iPSCs^{MYOD} or RC1–3. The protein expression level of hMYOGENIN in cell lysates were analyzed by western blotting on Day 8 (**d**). β-ACTIN was used as an internal control. Signal intensities were measured using ImageJ software. The protein expression level of hMYOGENIN (n = 30 each) were calculated after normalizing them to that of β-ACTIN and expressed relative to the levels in EDSSPD3-P1-iPSCs^{MYOD} on Day 8 (set as 1) (**e**). Data represent the mean ± SEM and are representative of ten independent experiments. **P < 0.01 versus EDSSPD3-P1-iPSCs^{MYOD}.

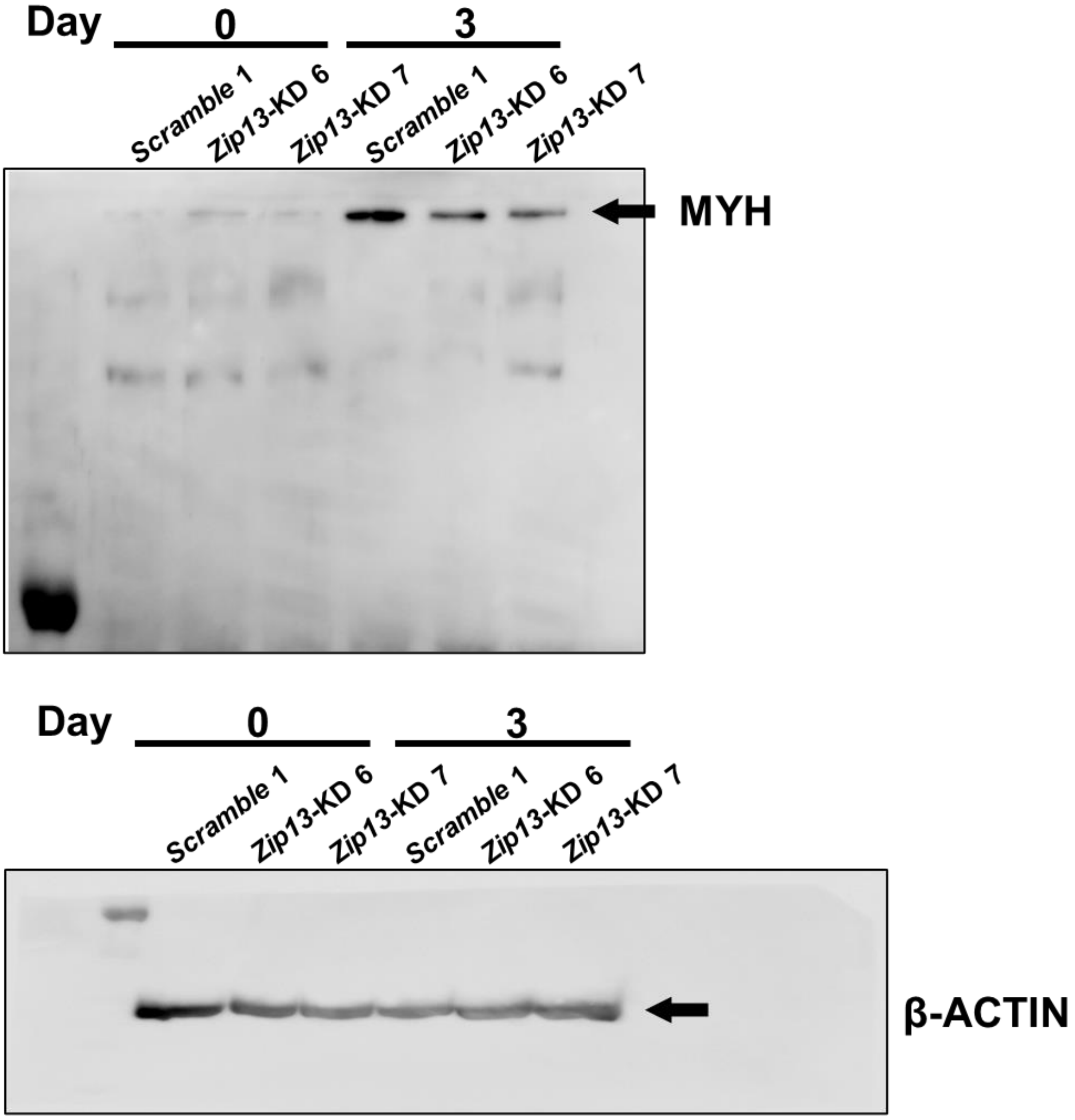

Supplementary Figure S10. Uncropped images of the western blotting membranes presented in Figure 1k. Uncropped full-length images of western blotting membrane of MYH protein expression in *Scramble* 1 and two clones of *Zip13*-KD (clone 6 and 7) of C2C12 cells during myogenic differentiation on Days 0 and 3. Black arrows showed protein bands recognized by indicated antibodies, and these bands are presented in Figure 1k.

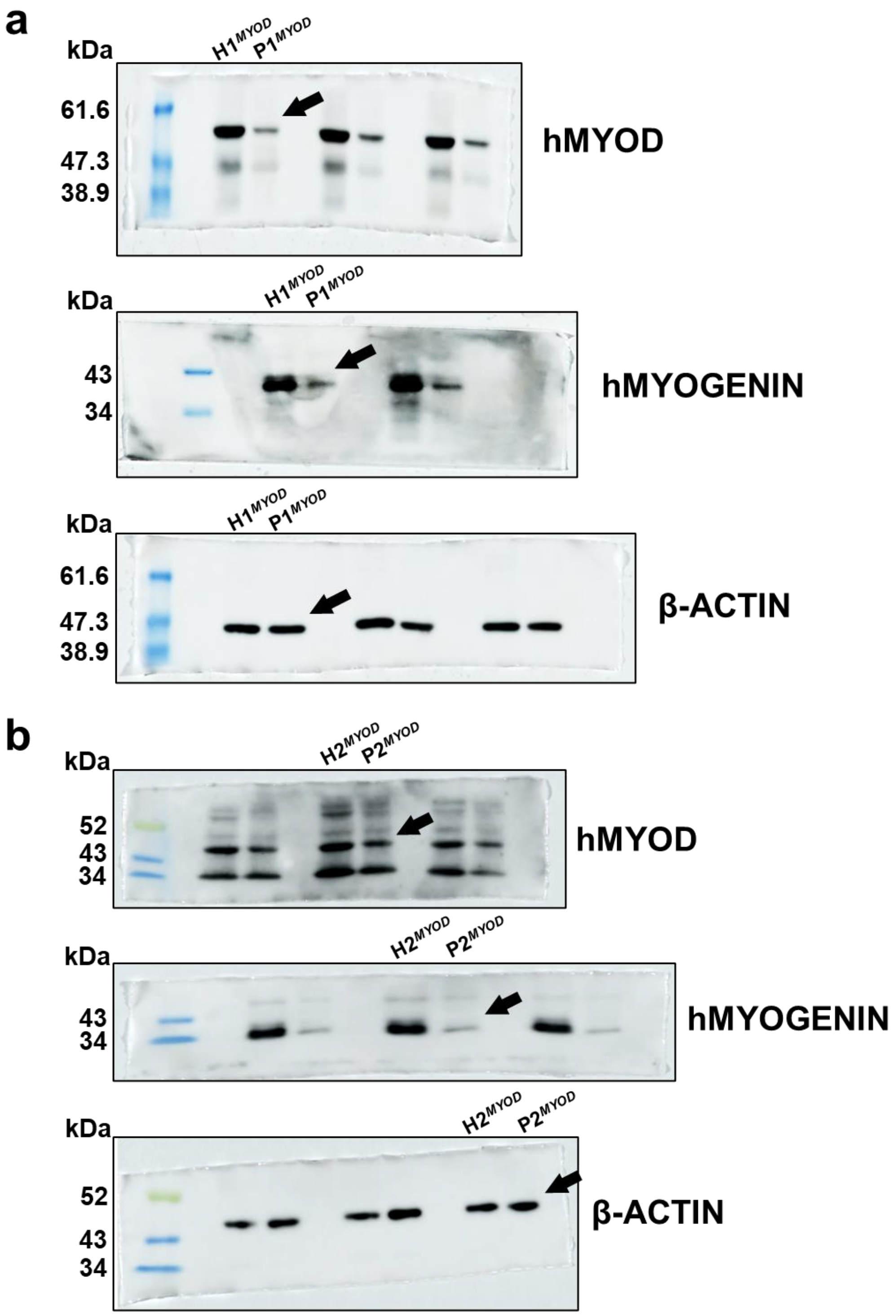

Supplementary Figure S11. Uncropped images of the western blotting membranes presented in Figure 3i and l. The black arrows indicate protein bands recognized by indicated antibodies, and these bands are presented in Figure 3i (**a**) and l (**b**). H1^{MYOD}: H1-iPSCs^{MYOD}, P1^{MYOD}: EDSSPD3-P1-iPSCs^{MYOD}, H2^{MYOD}: H2-iPSCs^{MYOD}, P2^{MYOD}: EDSSPD3-P2-iPSCs^{MYOD}.

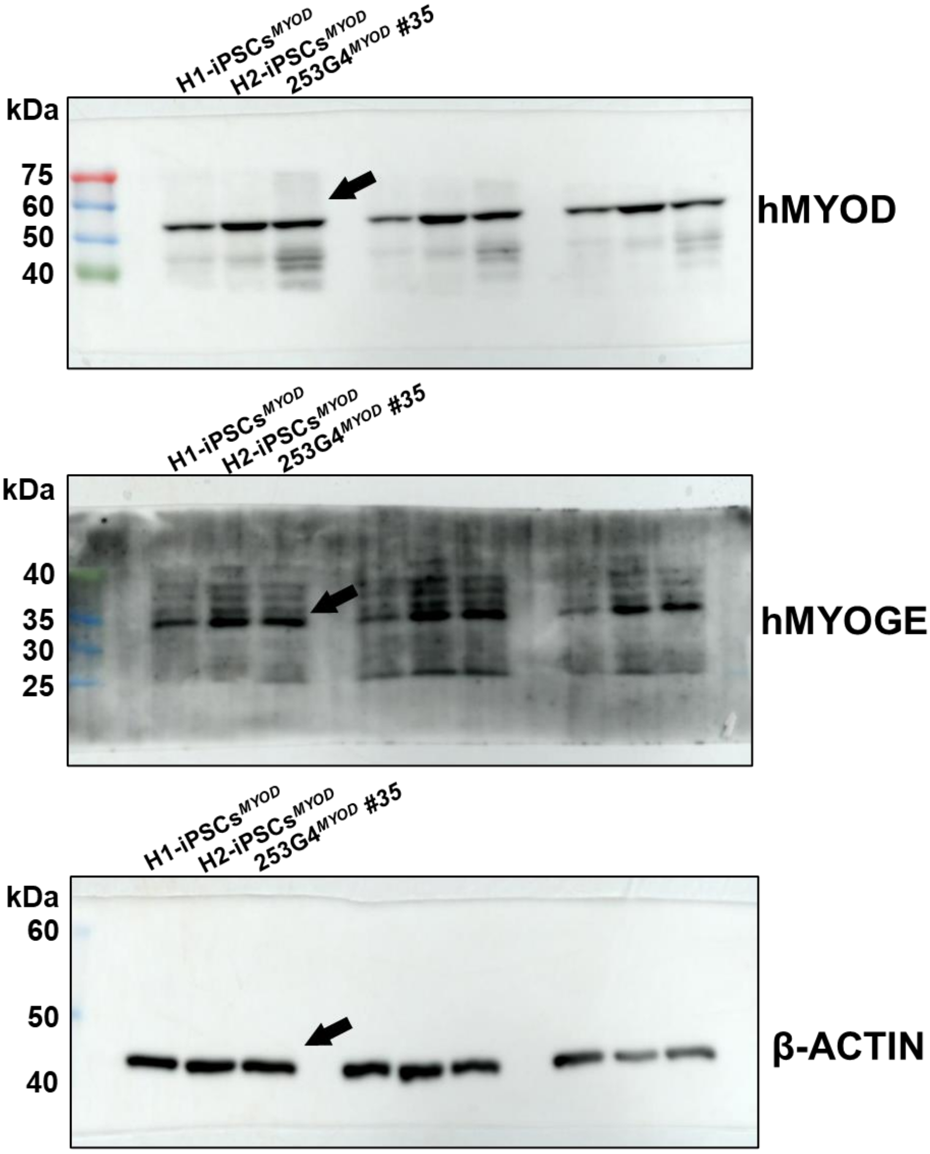

Supplementary Figure S12. Uncropped images of the western blotting membranes presented in Supplementary Figure S6h. The black arrows show protein bands recognized by indicated antibodies, and these bands are presented in Supplementary Figure S6h.

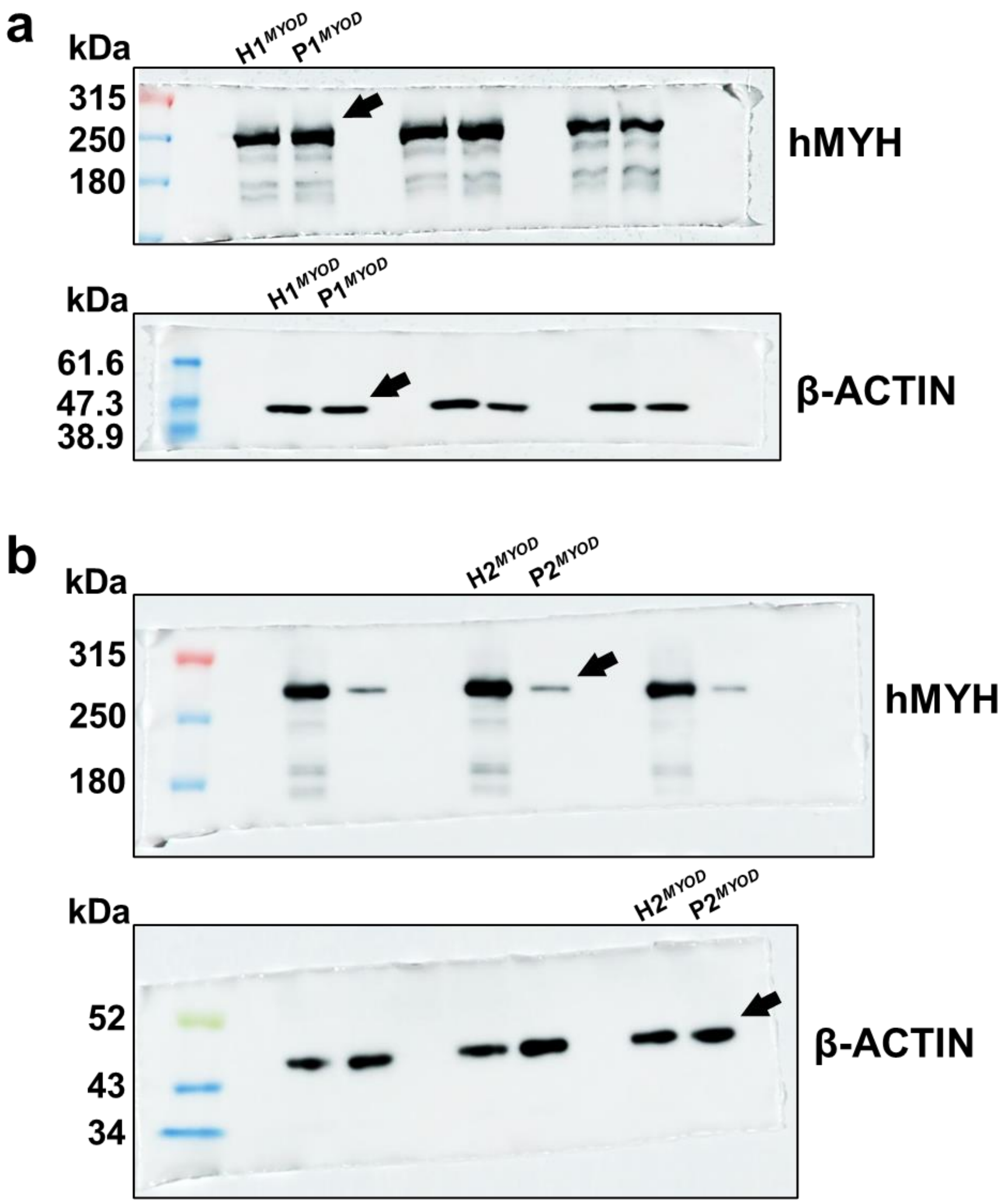

Supplementary Figure S13. Uncropped images of the western blotting membranes presented in Supplementary Figure S7p and r. The black arrows show protein bands recognized by indicated antibodies, and these bands are presented in Figure S7p (**a**) and r (**b**). H1^{MYOD}: H1-iPSCs^{MYOD}, P1^{MYOD}: EDSSPD3-P1-iPSCs^{MYOD}, H2^{MYOD}: H2-iPSCs^{MYOD}, P2^{MYOD}: EDSSPD3-P2-iPSCs^{MYOD}.

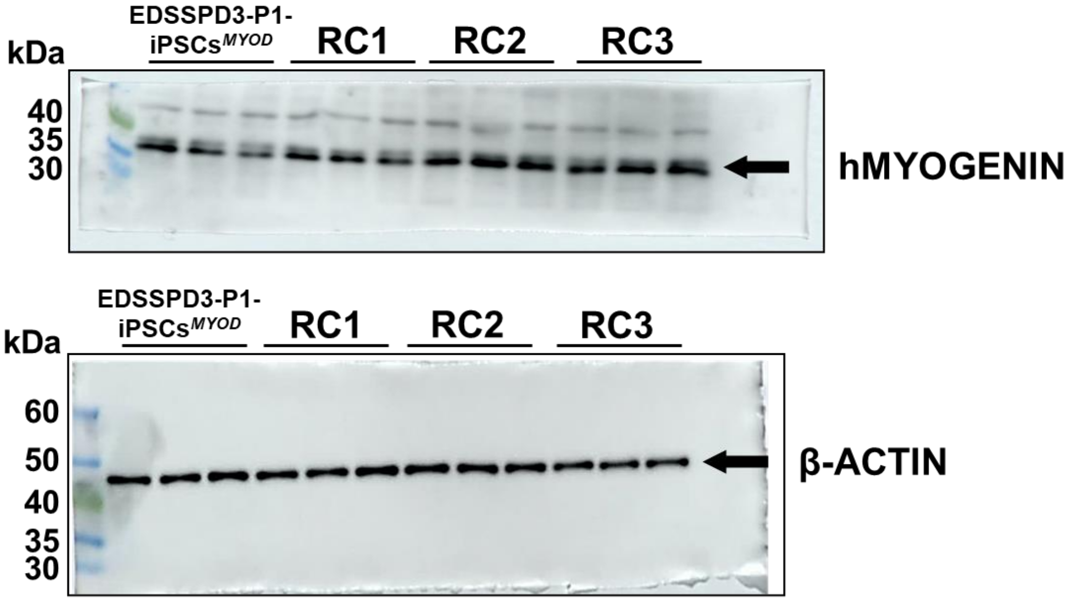

Supplementary Figure S14. Uncropped images of the western blotting membranes presented in Supplementary Figure S9d. The black arrows show protein bands recognized by indicated antibodies, and these bands are presented in Supplementary Figure S9d. RC1–3: three clones of *ZIP13*^{G64D}-corrected EDSSPD3-P1-iPSCs^{MYOD}.

Supplementary Table S1. List of the primers used in this study

**Target gene (symbol)/primer name**	**Sequences (Forward (F), Reverse (R); 5′ to 3′)**	**Experiment**
Scramble-shRNA	F: GATCCCCGTTACTTGAGCTCGTACGTTTCAAGAGAACGTACGAGCTCAAGTAACTTTTTA<br>R: AGCTTAAAAAGTTACTTGAGCTCGTACGTTCTCTTGAAACGTACGAGCTCAAGTAACGGG	Knockdown
mouse *Zip13*-shRNA	F: GATCCCCGCCTCACTTCTCATTGACAAATTCAAGAGATTTGTCAATGAGAAGTGAGGCTTTTTA<br>R: AGCTTAAAAAGCCTCACTTCTCATTGACAAATCTCTTGAATTTGTCAATGAGAAGTGAGGCGGG	Knockdown
mouse Zip13 (*mZip13*)	F: ACCGATGGACGGCAGCTAAG<br>R: AACACATTCACCAGGGCGATATAGA	RT-qPCR
mouse myogenic differentiation 1 (*mMyoD*)	F: CCCCGGCGGCAGAATGGCTACG<br>R: GGTCTGGGTTCCCTGTTCTGTGT	RT-qPCR
mouse Myogenin (*mMyogenin*)	F: GCAATGCACTGGAGTTCG<br>R: ACGATGGACGTAAGGGAGTG	RT-qPCR
mouse myogenic factor 5 (*mMyf5*)	F: CTGTCTGGTCCCGAAAGAAC<br>R: AAGCAATCCAAGCTGGACAC	RT-qPCR
mouse myosin heavy chain 2 (*mMyh2*)	F: AGGCGGCTGAGGAGCACGTA<br>R: GCGGCACAAGCAGCGTTGG	RT-qPCR
mouse β-actin (*β-actin*)	F: ACGGCCAGGTCATCACTATTG<br>R: CACAGGATTCCATACCCAAGAAG	RT-qPCR
mouse 18S ribosome	F: AGTCCCTGCCCTTTGTACACA<br>R: GATCCGAGGGCCTCACTAAAC	RT-qPCR

human ZIP13-exon2 mRNA	F: ATGGCGGGCCCAAGGCTCCTCTTCCT<br>R: TGTAGGCCCAGGCTTCGGGCAGCAGA	Sequencing analysis
human ZIP13-exon2 genome	F: CGTTTCCTAAGCAGGTGTGC<br>R: CTGTGACCTGGGGTCTCCTA	Sequencing analysis
human ZIP13-exon2 genome for In-Fusion_pGEM	F: CCGCGGGAATTCGATCGTTTCCTAAGCAGGTGTGC<br>R: GAATTCACTAGTGATCTGTGACCTGGGGTCTCCTA	In-Fusion cloning
mCherry	F: CATCCCCGACTACTTGAAGC<br>R: CCCATGGTCTTCTTCTGCAT	RT-qPCR
exogenous human myogenic differentiation 1 (*Exo-MYOD*)	F: CCCCTTCACCATGGAGCTA<br>R: AGTGCTCTTCGGGTTTCAGG	RT-qPCR
human ZIP13 (*hZIP13*)	F: CTACATCGCCTTGGTGAACGTG<br>R: AGTGAATCCGAGCATCTTATTGCTG	RT-qPCR
endogenous human myogenic differentiation 1 (*hEndo-MYOD*)	F: CACTCCGGTCCCAAATGTAG<br>R: TTCCCTGTAGCACCACACAC	RT-qPCR
human myogenin (*hMYOGENIN*)	F: TGGGCGTGTAAGGTGTGTAA<br>R: CGATGTACTGGATGGCACTG	RT-qPCR
human metallothionein 1H (*hMT1H*)	F: CAGCTGCTGTGCCTGATGTC<br>R: ATGTAGCAAATGAGTCGGAGTTGTA	RT-qPCR
human myogenic factor 5 (*hMYF5*)	F: CGCCTGAAGAAGGTCAACCA<br>R: ACATTCGGGCATGCCATCAG	RT-qPCR

human myosin heavy chain (*hMYH*)	F: GCAGATTGAGCTGGAAAAGG<br>R: TCAGCTGCTCGATCTCTTCA	RT-qPCR
human creatine kinase, M-type (*hCK-M*)	F: ACATGGCCAAGGTACTGACC<br>R: TGATGGGGTCAAAGAGTTCC	RT-qPCR
human glucose transporter type 4 (*hGLUT4*)	F: CCATCCTGATGACTGTGGCTCT<br>R: GCCACGATGAACCAAGGAATGG	RT-qPCR
human paired box 3 (*hPAX3*)	F: CCTCAGGTAATGGGACTCCTG<br>R: CCCCCTAAAAAGTCCAAGGCT	RT-qPCR
human paired box 7 (*hPAX7*)	F: GGAAGCGATTTTTGCCGACT<br>R: TTGTGGCGGATGTGGTTAGG	RT-qPCR
human housekeeping gene glyceraldehyde 3-phosphate dehydrogenase (*GAPDH*)	F: CGAGATCCCTCCAAAATCAA<br>R: CATGAGTCCTTCCACGATACCAA	RT-qPCR

Supplementary Table S2. Sequences of synthesized sgRNA and ssODN for correcting *ZIP13*^{G64D} mutation in iPSCs of patients with EDSSPD3 by the genome editing of HDR combined with CRISPR-Cas9 system

Name	Sequence (5′ to 3′)	
Scaffold-modified *ZIP13*^{G64D}_sgRNA	CUCCUGGAUUCCCUCAUGGU	sgRNA
Alt-R HDR-modified *ZIP13*^{G64D}-corrected_ssODN (143 mer)	CTGCGACGGCCTGTCGCCTGGACAACAAGGAAAGCGAGTCCTGGGGGGCT CTGCTGAGCGGAGAGCGGCTGGACACCTGGATCTGCTCCCTCCTGGGTTCA CTAATGGTGGGGCTCAGTGGGGTCTTCCCGTTGCTTGTCATT	ssODN

Supplementary Table S3. List of primary and secondary antibodies used in this study

Primary or Secondary	Target antigen	Host	Company, Catalog number	Experiment
Primary	Stage-specific embryonic antigen 4 (SSEA4)	Mouse	Stemgent, 09-0006	IF, FC
Primary	TRA-1-81	Mouse	Stemgent, 09-0011	IF, FC
Primary	Purified mouse IgG3 Isotype control	Mouse	BioLegend, 401301	FC
Primary	Myosin heavy chain (MYH)	Mouse	R&D Systems, MAB4470	IF
Primary	Myogenic differentiation 1 (MYOD)	Mouse	Santa Cruz Biotechnology, sc-377460	WB
Primary	Myogenin (MYOGENIN)	Mouse	Santa Cruz Biotechnology, sc-12732	WB
Primary	Myosin heavy chain (MYH)	Rabbit	Santa Cruz Biotechnology, sc-20641	WB
Primary	β-Actin	Rabbit	Cell Signaling, 4970	WB
Secondary	Alexa Fluor 488-conjugated anti-mouse IgG	Goat	Thermo Fisher Scientific, A11001	IF, FC
Secondary	Horseradish peroxidase-conjugated anti-mouse IgG1	Goat	SouthernBiotech, 1070-05	WB
Secondary	Horseradish peroxidase-conjugated anti-rabbit IgG	Goat	Sigma-Aldrich, A0545	WB

IF, immunofluorescence; FC, flow cytometry; WB, western blotting
